# Engineering Bipolar Doping in a Janus Dual-Atom Catalyst for Photo-Enhanced Rechargeable Zn-Air Battery

**DOI:** 10.1007/s40820-025-01707-2

**Published:** 2025-03-28

**Authors:** Ning Liu, Yinwu Li, Wencai Liu, Zhanhao Liang, Bin Liao, Fang Yang, Ming Zhao, Bo Yan, Xuchun Gui, Hong Bin Yang, Dingshan Yu, Zhiping Zeng, Guowei Yang

**Affiliations:** 1 State Key Laboratory of Optoelectronic Materials, School of Materials Science and Engineering, Sun Yat-Sen University, Guangzhou, 510275 People’s Republic of China; 2 Key Laboratory for Polymeric Composite and Functional Materials of Ministry of Education, Key Laboratory of High-Performance Polymer-Based Composites of Guangdong Province, School of Chemistry, Sun Yat-Sen University, Guangzhou, 510275 People’s Republic of China; 3 School of Materials Science and Engineering, Suzhou University of Science and Technology, Suzhou, 215009 People’s Republic of China; 4 State Key Laboratory of Optoelectronic Materials and Technologies, School of Electronics and Information Technology, Sun Yat-Sen University, Guangzhou, 510275 People’s Republic of China

**Keywords:** Janus dual-atom catalyst, Bifunctional center, Bipolar doping, Oxygen redox reaction, Fuel cell

## Abstract

Harnessing solar energy to enhance the rechargeable zinc–air batteries (RZABs) performance is a promising avenue toward sustainable energy storage and conversion. Simultaneously enhancing light-absorption capacity and carrier separation efficiency in nanomaterials, as well as improving electrical conductivity and configuration for electrocatalysis, presents a formidable challenge due to inherent trade-offs and interdependencies. Here, we have developed a Janus dual-atom catalyst (JDAC) with bifunctional centers for efficient charge separation and electrocatalytic performance through a bipolar doping strategy. The in situ X-ray absorption near-edge structure and Raman spectroscopy analyses demonstrated that the Ni and Fe centers in JDAC not only function as effective sites for oxygen evolution reaction and oxygen reduction reaction, respectively, but also serve as efficient hole and electron enrichment sites, effectively suppressing photoelectron recombination while enhancing photocurrent generation. As a result, the assembled JDAC-based light-assisted RZABs exhibited extraordinary stability at large current densities. This work delivers pivotal insight to design Janus dual-atom catalysts that efficiently convert solar energy into electric and chemical energy.

**Supplementary Information:**

The online version contains supplementary material available at 10.1007/s40820-025-01707-2.

## Highlights

- Janus dual-atom catalyst (JDAC) with bifunctional centers was synthesized via a single-step bipolar doping strategy to promote efficient charge separation and superior electrocatalytic performance.
- The in situ X-ray absorption near-edge structure and Raman spectroscopy analyses demonstrated that Ni and Fe centers in JDAC function as effective sites for oxygen evolution reaction and oxygen reduction reaction, and effectively suppress photoelectron recombination while enhancing photocurrent generation.
- The assembled JDAC-based light-assisted rechargeable zinc–air batteries exhibited extraordinary stability at large current densities (300 cycles at 50 mA cm^−2^, and 6000 cycles at 10 mA cm^−2^ under light illumination).

**Supplementary Information:**

The online version contains supplementary material available at 10.1007/s40820-025-01707-2.

## Graphical abstract

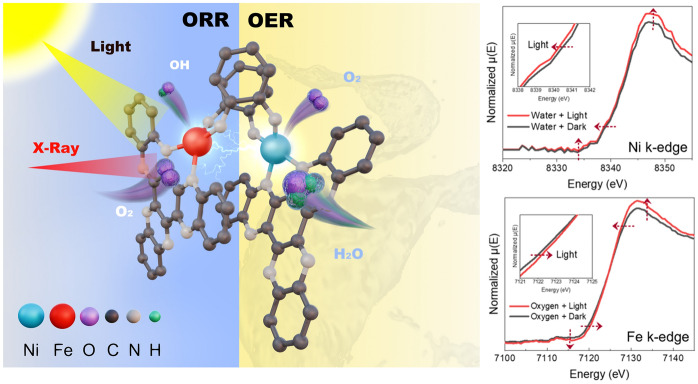

## Introduction

Rechargeable zinc–air batteries (RZABs) have gained much interest as a promising energy storage technology due to their remarkable theoretical energy density (1086 Wh kg^−1^), cost-competitiveness, environmental friendliness, and high safety [1, 2]. In recent years, solar-driven ZABs harness photogenerated holes and electrons to facilitate redox reactions, offering an attractive strategy for solar-driven energy storage and conversion. By harnessing the power of light, the abundant solar energy provides additional acceleration for catalytic reactions in solar-driven batteries [3]. It is essential for a photocathode that possesses both photoresponsivity and efficient catalytic activity for oxygen redox reactions in light-assisted RZABs. Current research on photocathode primarily concentrates on semiconductors such as BiVO_4_ [4], α-Fe_2_O_3_ [5], TiO_2_ [6, 7, 8], ZnO [9], poly(1,4-di(2-thienyl))benzene (PDTB) [10], polytrithiophene (pTTh) [11], and C_3_N_4_ [12]. However, these photocathodes exhibit a wide bandgap (2.3 eV for BiVO_4_, 2.2 eV for α-Fe_2_O_3_, 3.1 eV for TiO_2_, and 2.7 eV for g-C_3_N_4_), which constrains their ability to absorb visible light. C_4_N, a novel semiconductor with a narrow bandgap of 1.99 eV, induces a favorable photocoupling effect with visible-light response for enhanced oxygen catalysis reactions [13, 14]. However, another important aspect is that current photoelectrocatalysts commonly suffer from a high recombination rate of photogenerated electron–hole pairs, which is pronounced at high current densities. As a consequence, the assembled light-assisted RZABs are limited to operate at low current densities (0.1 or 1 mA cm^−2^). In addition, photoelectrocatalysts encounter challenges such as limited conductivity and insufficient inherent electrocatalytic activity [15]. To address these limitations, recent researches explored several strategies, such as constructing heterojunctions [16, 17], introducing chemical dopants [18], tailoring defects [19, 20], and utilizing piezoelectric fields [21, 22]. Evidently, the optimization of light-absorption capacity and carrier separation efficiency for photocatalysis, as well as enhancement of electrical conductivity and configuration of the catalytic sites for electrocatalysis, has become a crucial issue in designing solar-driven RZAB due to the distinct requirements for electrode materials in these two processes.

Single-atom catalysts (SACs) have emerged as highly promising photoelectrocatalyst candidates due to their ability to optimize both optical and electrochemical properties, which have been widely used as photoelectrocatalysts for various catalytic reactions [23–25]. Atomically dispersed metal atoms in photoelectrodes not only serve as centers for photogenerated carrier separation, acting as electron donor/acceptor sites [26] or electron-withdrawing sites [27], but also reduce the bandgap and introduce additional mid-gap defect states. These features enhance light-harvesting capability, suppress charge recombination, and extend the carrier lifetime during photocatalysis [28, 29]. Moreover, the variety of single atoms with diverse coordination environments in photoelectrodes endow the catalyst with distinct active sites and increased electronic conductivity via π-electron modulations, thereby enhancing electrocatalytic performance [30–32]. Fe-N_4_ and Ni-N_4_ sites confer outstanding electrocatalytic activity for oxygen reduction and oxidation reactions [2, 33–36]. Although efforts have been made to integrate SACs on semiconductors, SAC-based photoelectrodes with bifunctional oxygen catalysis remain unexplored. Developing bifunctional SAC-based photoelectrode is of great significance for overcoming the challenges of inefficient charge transfer and severe carrier recombination, as well as for gaining a clear understanding of mechanisms for photo-enhanced bifunctional oxygen catalysis.

Herein, we rationally designed a Janus dual-atom catalyst (JDAC) via a one-step hydrothermal strategy. In situ X-ray absorption near-edge structure (XANES) and Raman spectroscopy analyses demonstrated that Ni and Fe centers in JDAC serve as effective hole and electron enrichment sites, effectively suppressing photoelectron recombination and enhancing photocurrent generation. Moreover, the enrichment of holes on Ni sites and electrons on Fe sites in JDAC further facilitates the electrocatalytic oxygen evolution reaction (OER) and oxygen reduction reaction and (ORR). The assembled light-assisted rechargeable RZAB equipped with a JDAC-based cathode exhibits extraordinary stability at large current densities (300 cycles at 50 mA cm^−2^, 1600 cycles at 20 mA cm^−2^, and 6000 cycles at 10 mA cm^−2^ with negligible voltage decay under light illumination). This rational Janus dual-atom catalyst facilitates the development of light-assisted RZABs with effective utilization of solar energy.

## Experimental Section

### Synthesis of JDAC, Fe-C_4_N, Ni-C_4_N, and C_4_N

The JDAC is synthesized via in situ coupling of hexaketocyclohexane (HKH), 3,3′,4′4-biphenylteramine (BPTA), Fe(AC)_2_, and Ni(AC)_2_. The above precursors were dissolved in a 25-mL solution composed of an equal volume of methanol and 1-Methyl-2-pyrrolidinone (NMP), and put into a 50-mL hydrothermal autoclave reactor along with carbon paper for hydrothermal reaction at 175 °C for 12 h. The JDAC on carbon paper was then dried at 60 °C for 24 h. JDAC powders were collected by vacuum filtrating the above solution after hydrothermal reaction. To obtain in situ grown Fe-C_4_N, Ni-C_4_N, and C_4_N, a similar process was implemented as the fabrication of JDAC. The precursors for Fe-C_4_N were HKH, BPTA, and Fe(AC)_2_, while Ni-C_4_N included HKH, BPTA, and Ni(AC)_2_. The precursors for C_4_N were HKH, BPTA.

### Optical Properties Measurement

To determine the bandgap structure of the as-prepared samples, UV–Vis spectra and XPS-VB spectra were measured via a UV–Vis spectrophotometer (METASH, UV-5200PC), and a X-ray energy spectrometer (XPS, ESCALab 250), respectively. The femtosecond transient absorption spectroscopy (fs-TAS) was measured through a femtosecond–nanosecond transient absorption spectrometer (Helios Fire) with a pump wavelength of 600 nm. Mott–Schottky plots of the as-prepared samples were measured in the dark at various frequencies using a CHI 760 electrochemical workstation (CH Instruments, Inc., Shanghai). The carrier densities of JDACs and other catalysts were calculated based on the Mott–Schottky equation:

$$\frac{1}{{C_{{{\text{SC}}}}^{2} }} = \frac{2}{{{\text{Ne}}_{0} \varepsilon_{0} \varepsilon_{r} }}\left( {E - E_{{{\text{FB}}}} - \frac{kT}{e}} \right)$$

### Electrochemical Measurement

The electrochemical tests were carried out using CHI 760 electrochemical workstation (CH Instruments, Inc., Shanghai) with a standard three-electrode configuration at room temperature. The ORR measurements were conducted in O_2_-saturated 0.1 mol L^−1^ KOH solution with a scan rate of 10 V s^−1^, while OER measurements were conducted in 1 mol L^−1^ KOH solution. All electrochemical tests under illumination were conducted with a visible-light simulator (Xenon lamp source, PLS-SXE300 + , equipped with a 420 nm-cut filter, UVCUT 420/φ63 × 4 mm).

### Assembly of Light-Assisted Rechargeable Zn-Air Battery

The Zn-air battery is assembled by a two-electrode configuration, with the carbon paper containing corresponding electrocatalyst as the air cathode and Zn plate (0.5 mm) as the anode. 6 mol L^−1^ KOH + 0.2 mol L^−1^ Zn(AC)_2_ aqueous solution was used as the electrolyte, and a solar-light simulator (Xenon lamp source, PLS-SXE300 + , equipped with an AM 1.5 G filter, AM 1.5 G/φ63 × 4 mm) was used for illumination of the air cathode.

### Theoretical Calculation

Gibbs free energies for the process were calculated based on t $\Delta G = \Delta E + \Delta {\text{ZPE}} - T\Delta S,$ where Δ*E*, ΔZPE, and Δ*S* are the total energy, zero-point energy, and entropy change relative to the initial state. ZPE and TS are the zero-point energy and entropy contributions, calculated from the vibrational frequencies, T represents the temperature (298.15 K). Here, the equilibrium potential U_0_ for the ORR was determined to be 1.23 V vs. RHE. The free energy of the O_2_ molecule is achieved based on $G_{{{\text{O}}_{2} \left( g \right)}} = 2G_{{{\text{H}}_{2} {\text{O}}\left( l \right)}} - 2G_{{H_{2} }} + 4 \times 1.23 \left( {{\text{eV}}} \right).$ All density functional theory (DFT) [37, 38] calculations were taken by using the Gaussian 16 C.01 program [39]. The geometry optimizations were carried out using the PBE0 [40] functional and a def2-SVP [41, 42] basis set in the gas phase. In addition, the D3 version of Grimme’s dispersion correction with the original D3 damping function was considered in structure optimizations and energy calculations [43]. The 3D optimized structures were visualized via the CYLview [44] visualization programs. The molecular orbitals and natural bond orbitals were depicted using the IQmol [45] program (Isovalue = 0.02). The final overpotentials for OER and ORR were calculated by the difference between the Gibbs free energy change and equilibrium potential (1.23 V) as [46]:${\upeta }_{{{\text{OER}}}} = {\text{max}}\left[ {{\Delta }G_{1,2,3,4} } \right]/e - 1.23\;{\text{V}}.$

## Results and Discussion

### Design and Structure Characterizations

**Fig. 1:**
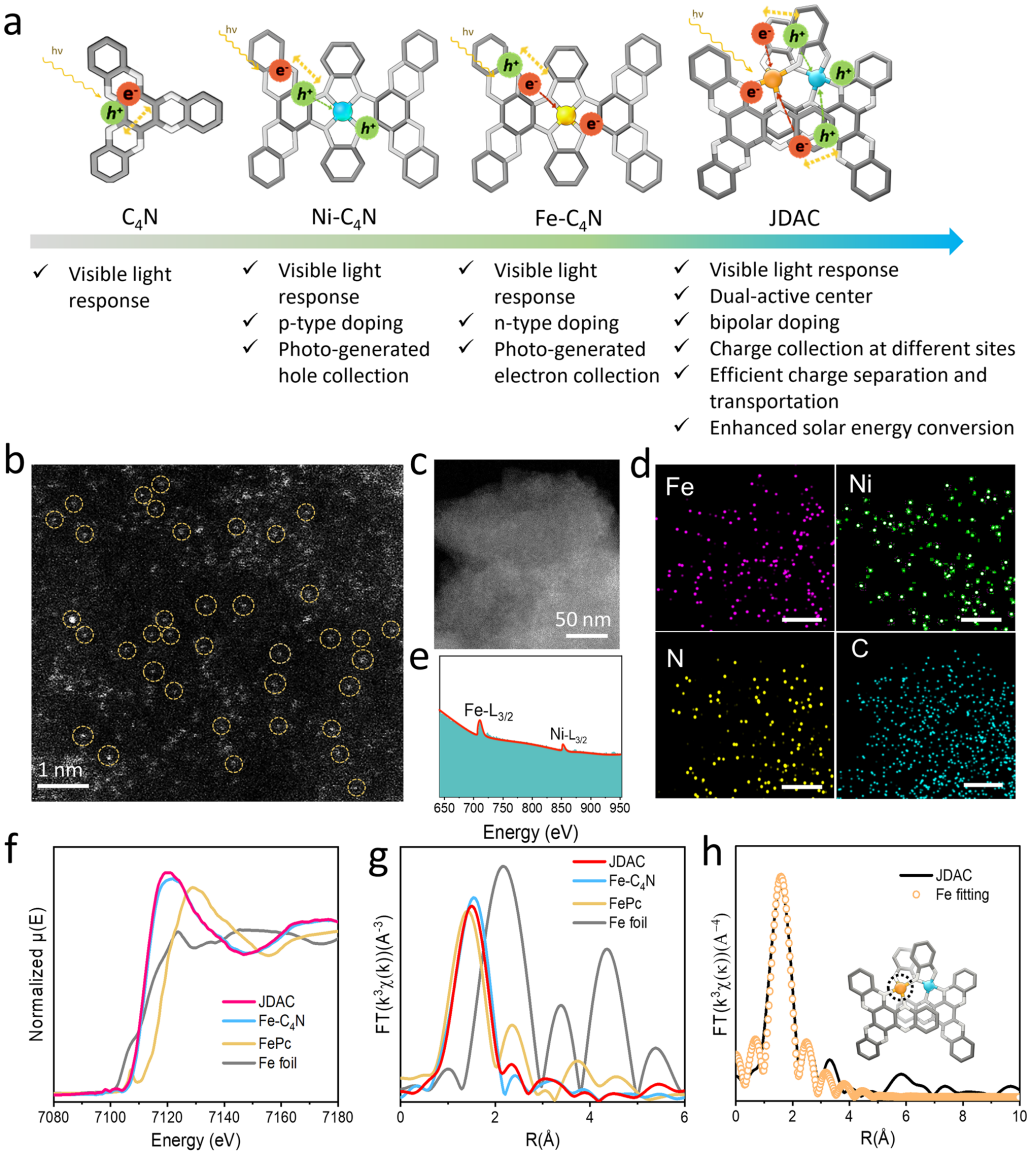
**a** Schematic illustration of the advantages of JDAC. **b** Aberration-corrected HAADF-STEM image of JDAC with single atom being highlighted by yellow circle. **c** High-resolution STEM image. **d** EDS elemental mapping (scale bar 50 nm). **e** Electron energy loss spectrum (EELS) of JDAC. **f** XANES spectra of Fe K-edge. **g** Fourier transformation-EXAFS and **h** EXAFS fitting curves of Fe in JDAC

The dual-atom catalyst JDAC featuring dual-atom centers, bipolar dopants, and efficient photogenerated charge separation and transfer (Fig. 1a) was synthesized on carbon paper through the in situ coupling of hexaketocyclohexane (HKH), 3,3′, 4′,4-biphenylteramine (BPTA), ferric acetate, and nickel acetate (Fig. S1). The aberration-corrected high-angle annular dark-field scanning transmission electron microscopy (HAADF-STEM) image reveals distinct and well-dispersed bright dots, indicating the presence of single atoms (Figs. 1b and S2). Elemental mapping shows a uniform dispersion of Fe, Ni, C, and N throughout the catalyst (Fig. 1c, d). Additionally, electron energy loss spectroscopy (EELS) result displays two prominent peaks, corresponding to Fe and Ni, confirming their existence in the catalyst (Fig. 1e). Scanning electron microscope (SEM) images show that the surface topography of in situ grown JDAC on carbon paper exhibits a highly porous structure (Fig. S3). Fourier transform infrared spectra (FT-IR) reveal that the carbonyl (C=O) peak at 1623 cm^−1^, characteristic of HKH, and the amino (–NH_2_) peak at 3343 cm^−1^, associated with BPTA, have been converted to the C=N peak at 1592 cm^−1^ in both C_4_N and JDAC (Fig. S4). The nuclear magnetic resonance spectrum (NMR) spectra for C_4_N and JDAC exhibit distinct resonances at 130 and 142 ppm (Fig. S5), which are attributed to the C=C bonds in the benzene rings and the C=N bonds in the pyrazine rings, respectively [47]. These experimental outcomes confirm the fabrication of the C_4_N structure in JDAC. XPS was used to study the elemental chemical states of JDAC (Figs. S6–S10). In the high-resolution N 1*s* XPS spectra of samples (Figs. S7–S9), the dominant N peak at 400.0 eV attributes to metal–N binding [48], while the weak peak at 398.8 eV corresponds to pyrazine-N [49]. The N 1*s* spectra in C_4_N (Fig. S10b) show a pyrazine-N peak at 398.8 eV and a weak peak at ~ 399.9 eV, indicative of residual –NH_2_ groups [50]. This change in peak intensity suggests chemical bonding between the nitrogen atoms in C_4_N and the metal single atoms. High-resolution Fe 2*p* spectrum of JDAC exhibits a typical Fe 2*p*_1/2_ (723.9 eV) and Fe 2*p*_3/2_ (710.2 eV) doublet accompanied by two satellite peaks, and the Ni 2*p* spectrum shows two characteristic peaks of Ni 2*p*_1/2_ (873.5 eV) and Ni 2*p*_3/2_ (855.6 eV) orbitals with two corresponding satellite peaks (Fig. S7) [23]. It can be seen that the electron binding energy of Fe 2*p* and the Ni 2*p* orbitals in JDAC displays a certain degree of negative shift compared with Fe-C_4_N and Ni-C_4_N (Fig. S11). Such shift of the binding energies may originate from electronic interactions between Fe and Ni in JDAC [24]. X-ray diffraction (XRD) analysis exhibits no crystalline peaks corresponding to metals or metal oxides (Fig. S12), providing further evidence for the atomic distribution of Fe and Ni. As indicated by inductively coupled plasma atomic emission spectroscopy (ICP-AES), the Fe and Ni metal contents in JDAC are 7.96 and 6.52 wt% (Table S1), respectively. Furthermore, N_2_ adsorption–desorption analysis shows that JDAC exhibits a larger specific surface area as compared to Fe-C_4_N and Ni-C_4_N (Figs. S13–S15).

X-ray absorption spectroscopy (XAS), which includes XANES and extended X-ray absorption fine structure (EXAFS), serves as a valuable analytical tool for elucidating the detailed local coordination and valence states of metal atoms. The Fe K-edge XANES spectra of JDAC and Fe-C_4_N exhibit adsorption edges that fall between those of Fe foil and FePc (Fig. 1f). This suggests that the oxidation state of Fe in both JDAC and Fe-C_4_N is in between 0 and + 2. Moreover, the Ni K-edge XANES spectra exhibit that the adsorption edges of Ni in JDAC and Ni-C_4_N locate between those of Ni foil and NiPc, suggesting that the oxidation state of the Ni atoms lies within the range of 0 to + 2 (Fig. S16a). The Fourier transform *k*^2^-weighted extended X-ray absorption fine structure (FT-EXAFS) analysis of Fe displays prominent peaks at 1.48 Å in JDAC and 1.54 Å in Fe-C_4_N, attributed to the first shell scattering between Fe and N (Fig. 1g). Notably, the metal–metal scattering path at around 2.17 Å is absent in these samples, indicating atomic dispersion of Fe atoms with no Fe–Ni bonding. JDAC and Ni-C_4_N exhibit similar Ni–N scattering peaks at ~ 1.5 Å, whereas the metal–metal scattering peak at ~ 2.09 Å is absent, conveying information on atomic dispersion of Ni with no Ni–Fe bonding (Fig. S16b). EXAFS fitting results reveal that Fe and Ni atoms in JDAC have a coordination number of 3.80 and 4.22, respectively (Fig. 1h and Table S2), demonstrating a stable four-nitrogen coordination structure (Fe-N_4_ and Ni-N_4_). Wavelet transform (WT)-EXAFS of the *k*^2^-weighted EXAFS of Fe and Ni was further analyzed for estimation of the metal–nitrogen paths. As shown in Fig. S17, the intensity maxima of Fe K-edge reference in JDAC are positioned at 4.35 Å^−1^, identical to that of FePc, showing the presence of Fe–N bonds. Similarly, the Ni K-edge reference of JDAC exhibits an intensity maximum, which is identical to NiPc (Fig. S18). No obvious metal bond signals appear in the WT signal of JDAC, indicating no metal bonding conditions, which is in congruence with FT-EXAFS results. These findings further confirm that the Fe and Ni in the JDAC catalyst are mainly distributed as single atoms with metal–N_4_ paths, instead of aggregating into metal clusters or nanoparticles with metal–metal bonds.

### Photoelectric Properties of JDAC Cathodes

**Fig. 2:**
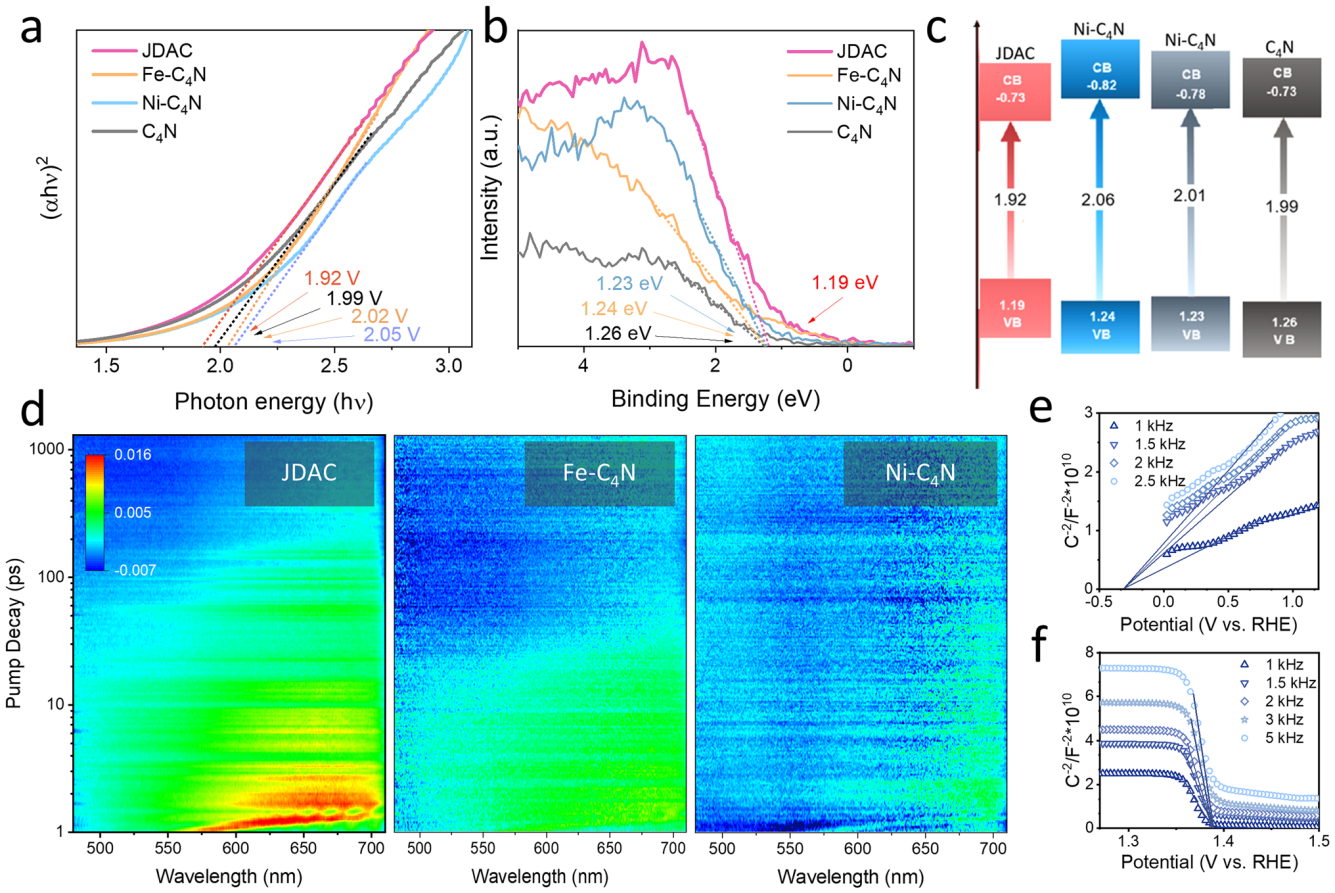
Bandgap characterization and spectroscopic analysis. **a** The Tauc plot and **b** XPS valence band spectra of JDAC, Fe-C_4_N, Ni-C_4_N, and C_4_N. **c** Schematic illustration of the bandgap and proposed working mechanism. **d** Femtosecond transient absorption spectroscopy (*fs*-TAS) of samples with pump at 600 nm. The Mott–Schottky plots of C_scL_^−2^ *vs.* E with **e** positive and **f** negative slopes for the JDAC in two potential regions

The photoelectron excitation and photoelectron transport properties of dual-atom-doped photoelectrode were further investigated using UV–Vis absorption spectra (UV–Vis) and femtosecond transient absorption spectra (fs-TAS). JDAC, Fe-C_4_N, and Ni-C_4_N exhibit comparable absorption profiles within the wavelength range of 300–900 nm, presenting strong absorption of visible light (Fig. S19). A notable difference can be observed in the 400–700 nm wavelength region, where JDAC generally displays a higher absorption intensity than C_4_N. Based on the Tauc plot derived from the UV–Vis spectrum, the bandgap (*E*_g_) of JDAC was calculated to be 1.92 eV (Fig. 2a), which is smaller than that of Fe-C_4_N (2.02 eV), Ni-C_4_N (2.05 eV), and C_4_N (1.99 eV). This confirms that the integration of dual atoms into C_4_N introduces additional energy levels, which creates impurity levels to narrow the bandgap and extend the range of light absorption [51]. The valance band X-ray photoelectron spectroscopy (VB-XPS) shows that the valence band of C_4_N is at 1.25 eV (Fig. 2b), which is close to the previously reported value [33]. In contrast, the valence band of JDAC is located at 1.19 eV, suggesting that the introduction of dual atoms alters the electronic structure. As illustrated in Fig. 2c, the narrow bandgap and favorable valence band energy endow JDAC with the capability to harness visible light and generate photo-induced holes and electrons suitable for ORR and OER catalysis. Femtosecond transient absorption spectroscopy (fs-TAS) measurements indicate that upon excitation at 600 nm, JDAC exhibits characteristic decay dynamics of photogenerated carrier in the range of 550–700 nm (Fig. 2d). JDAC displays a higher excitation intensity and an extended carrier lifetime relative to Fe-C_4_N, Ni-C_4_N, and C_4_N (Figs. 2d and S20). This superior charge separation and transfer in JDAC under visible-light irradiation are crucial for the photoelectrocatalytic process. We further investigated the effect of implanted metal dual atoms on C_4_N catalyst carrier concentration using Mott–Schottky measurements (Figs. 2e, f and S21). The plots display the relationship between the inverse square of the capacitance (C_scL_^−2^) and the electrode potential (E) [52]. Notably, the data reveal both positive and negative slopes for the JDAC within two distinct potential regions. This observation confirms the coexistence of n-type and p-type doping domains in the JDAC samples, indicative of their bipolar doping characteristics. In contrast, the Mott–Schottky plots of Fe-C_4_N and Ni-C_4_N exhibit single-slope characteristics, suggesting n-type and p-type doping of Fe-C_4_N and Ni-C_4_N, respectively (Fig. S21). The slope of Mott–Schottky plot of the n-type region derived from the slope in JDAC (1.37 × 10^10^ C^−2^ V) is higher than those of Fe-C_4_N (3.00 × 10^10^ C^−2^ V) and C_4_N (3.54 × 10^10^ C^−2^ V). Consistently, in the p-type region, the Mott–Schottky slope of JDAC (− 8.70 × 10^11^ C^−2^ V) is larger as compared to Ni-C_4_N (− 11.44 × 10^11^ C^−2^ V). According to Mott–Schottky equation, the carrier density is inversely proportional to the Mott–Schottky slope. Thus, JDAC possesses largest carrier density compared to Fe-C_4_N, Ni-C_4_N, and C_4_N. This enhanced carrier behavior is ascribed to the altered local electron environment induced by Fe and Ni atoms, which facilitate exciton separation, extend carrier lifetime and increase carrier density during photoelectrocatalysis. Moreover, JDAC exhibited a stable photocurrent value, which is the largest among Fe-C_4_N, Ni-C_4_N, and C_4_N, indicating good photoelectric response and photo-stability (Fig. S22).

### Light-Assisted Electrocatalytic Performance

**Fig. 3:**
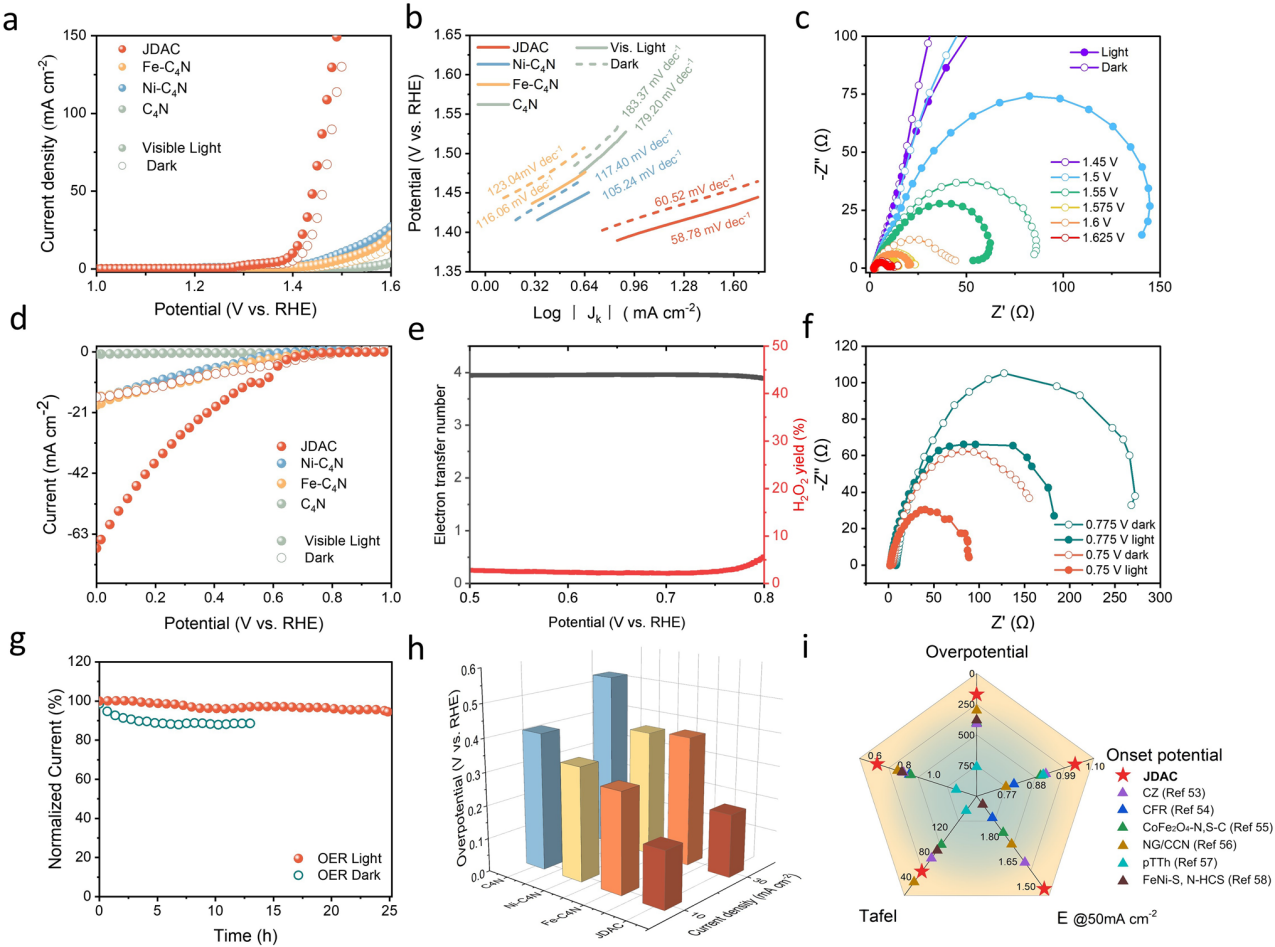
**a** Linear sweep voltammograms (LSVs) with and without visible-light irradiation. **b** Tafel slopes for OER. **c** Electrochemical impedance spectroscopy (EIS) during the OER process. **d** LSV curves with and without visible-light irradiation. **e** Electron transfer number and H_2_O_2_ selectivity of JDAC during the ORR process. **f** EIS during the ORR process. **g** Stability measurement of JDAC in 0.1 M KOH solution with saturated O_2_ and under a bias voltage of 1.4 V (vs. RHE). **h** Histogram of $E_{{10\;{\text{mA}}\;{\text{cm}}^{-2} }}$ and $E_{{20\;{\text{mA}}\;{\text{cm}}^{-2} }}$. **i** Comparison of overpotential, onset potential (E@0.01 mA cm^−2^), potential at 50 mA cm^−2^ (E@50 mA cm^−2^), OER Tafel slopes, and the potential gap ΔE (E_j=10_—E_1/2_) of different samples [53, 54–57, 58]

Linear sweep voltammetry (LSV) was used to investigate the effects of Fe and Ni dual-atom implantation on the electrocatalytic properties of C_4_N materials and their photocatalytic properties. The OER activity of the JDAC catalyst achieved an excellent overpotential (η) of 170 mV at 10 mA cm^−2^ under visible light, much lower than that of Fe-C_4_N (310 mV), Ni-C_4_N (270 mV), and C_4_N (410 mV) (Fig. 3a). JDAC also exhibited a remarkably low Tafel slope of 58.78 mV dec^−1^ under visible-light illumination, indicating its superior electron transfer kinetics (Fig. 3b). As shown in Figs. 3d and S23, the ORR catalytic performance of JDAC can be significantly enhanced upon visible-light illumination. JDAC reached a current density of 20 mA cm^−2^ at a potential of 0.35 V (vs. RHE). Without illumination, JDAC has only a current density of 7.7 mA cm^−2^ at the same potential, showing relatively poor ORR activity. JDAC exhibits a relatively small Tafel slope for ORR compared to other samples (Fig. S24). The LSV plots under visible light, along with the corresponding Koutecky–Levich (K–L) results, indicate that JDAC exhibits a favorable four-electron transfer pathway (Figs. S25 and S26). Notably, based on rotating ring disk electrode (RRDE) measurements, the H_2_O_2_ yield of JDAC remains below 5% across the potential range of 0.5 to 0.8 V (vs. RHE) (Figs. 3e and S27). Electrochemical impedance spectroscopy (EIS) measurements were performed at 1.45–1.625 V (vs. RHE) for OER (Fig. 3c) and 0.75–0.775 V (vs. RHE) for ORR (Fig. 3f). It can be observed that JDAC possesses a much smaller electron transfer resistance (*R*_ct_) than raw C_4_N, and the visible-light illumination significantly decreases the *R*_ct_ of JDAC. The photoelectrocatalytic stability measurements revealed that JDAC exhibited remarkable durability, with the OER current retention of 97% after 25 h at 1.65 V (vs. RHE) under visible-light irradiation (Fig. 3g), and the ORR current remained stable for over 50 h at 0.65 V (vs. RHE) (Fig. S28). SEM, XRD, and XPS results indicate that the structure and composition of JDAC remain unchanged after cycling stability tests (Figs. S29–S32). Benefiting from the well-designed Janus dual-atom sites, the OER and ORR activities of JDAC outperform Fe-C_4_N, Ni-C_−4_N, C_4_N, and other previously reported photoelectrocatalysts (Fig. 3h, i and Table S3).

### In Situ Elucidation of the Operational Mechanism

**Fig. 4:**
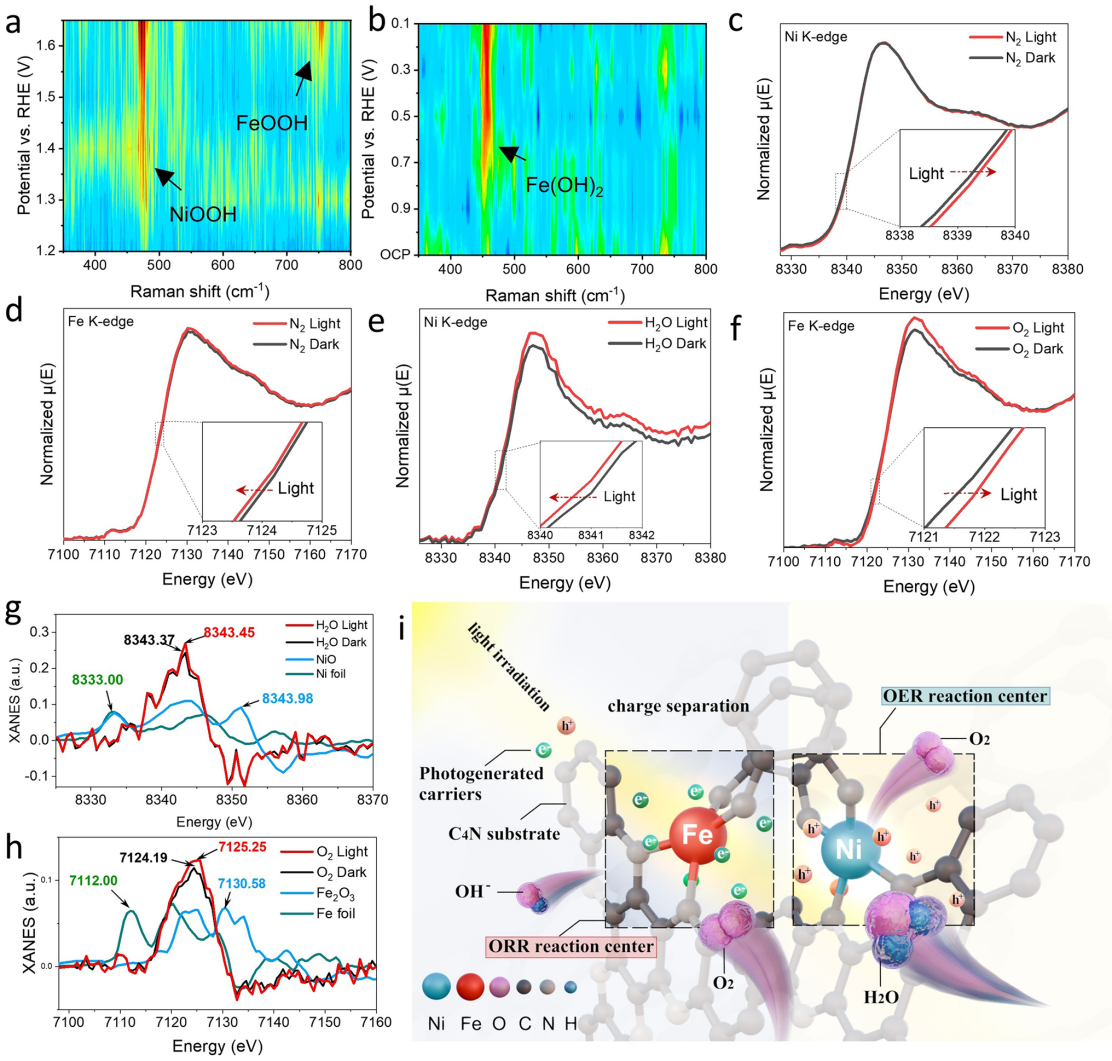
In situ Raman spectra of JDAC during **a** OER and **b** ORR process. In situ XANES of **c** Ni and **d** Fe K-edge in N_2_ with and without visible-light illumination. The insets depict magnified images at the near-edge region. **e** In situ XANES of Ni K-edge in H_2_O with and without visible-light illumination. **f** The first-derivate XANES plots of Ni K-edge. **g** In situ XANES of Fe K-edge in O_2_ with and without visible-light illumination. **h** The first-derivate XANES plots of Fe K-edge. **i** Schematic illustration of the operational mechanism of the JDAC with efficient hole and electron enrichment sites for photo-enhanced ORR and OER

To investigate the underlying mechanism of bifunctional oxygen redox mechanism, in situ Raman and XANES analysis was performed to elucidate the active sites involved in the OER and ORR. The in situ Raman spectra were recorded in a 1 M KOH electrolyte over the OER voltage range of 1.1 to 1.7 V vs. RHE (Figs. 4a and S33). At the applied potentials of 1.1 and 1.2 V, no characteristic peak was observed because OER did not occur. Once the potential was switched to 1.3 V and even larger, one obvious peak appears at 472 cm^−1^, which is attributed to Ni-OOH [59]. Upon increasing the applied voltage to 1.55 V, an additional weak peak appears at 749 cm^−1^, which corresponds to Fe-OOH [60]. This finding suggests that the oxygen intermediates of OER initially accumulate on Ni sites prior to the activation of Fe sites. Concerning the ORR, in situ Raman spectra were conducted over the potentials from 0.9 to 0.1 (vs. RHE) in a 0.1 M KOH electrolyte (Fig. 4d). In the initial state with no applied voltage, no distinct Raman features were recorded from 350 to 1200 cm^−1^, indicative of the absence of ORR. When the potential was increased to 0.85 V, a distinct peak appears at 456 cm^−1^, which is ascribed to Fe(OH)_2_ [61]. As the potential is gradually decreased from 0.8 to 0.1 V, the Raman peak at 456 cm^−1^ exhibits a slight increase in intensity, suggesting that the concentration of oxygen intermediates on Fe sites is rising. Nevertheless, the characteristic peak at 472 cm^−1^ for Ni-OOH cannot be clearly observed. These findings verify that Fe, rather than Ni, is the preferred active sites for ORR.

Using in situ XANES spectroscopy, we first investigate the effect of Ni and Fe dual-atoms incorporation on the distribution of electrons and holes produced by photoexcitation in JDAC catalysts. As shown in Fig. 4b, under illumination, the absorption edge of Ni in JDAC exhibited a rightward shift (red arrows), indicating an increase of oxidation state, could be ascribed to the accumulation of photogenerated holes. However, under the same conditions, we observe that the absorption edge of Fe moves toward the lower energy (Fig. 4e), indicating that its oxidation number decreases, which is attributed to the enrichment of photogenerated electrons on Fe atoms. It can be observed that the introduction of Ni and Fe atoms forms a photogenerated hole and electron enrichment center, which greatly improves the activity of OER and ORR. Considering that the reactants for the OER and ORR reactions are H_2_O (OH^−^) and O_2_, respectively, we further investigated the interaction between Ni and H_2_O, as well as Fe sites and O_2_ molecules in JDAC under light illumination. It can be seen that when H_2_O is present, the oxidation number of Ni decreases compared with that of no reactants, adsorption edge shift to lower energy side approximately 0.2 eV (Fig. 4c), indicating that electrons transfer from OH^−^ in H_2_O to the Ni active site, which is consistent with the conclusion that OER-adsorbed reactant OH^−^ is more likely to occur under higher hole density at the Ni site under light illumination (Fig. 4g). In contrast, the absorption edge of Fe in JDAC exhibited no obvious shift when exposed to H_2_O under light illumination (Fig. S34a). Moreover, in the presence of O_2_, the absorption edge of Fe in JDAC showed a shift of approximately 0.4 eV to higher energy side (Fig. 4f, h), indicating electron transfer from Fe to O_2_, which could be ascribed to the bond formation between Fe and O_2_. Meanwhile, no obvious shift appears in the absorption edge spectra of Ni in JDAC upon exposure to oxygen and visible-light irradiation (Fig. S34b). By conducting in situ Raman and XANES analysis, it has been observed that Ni and Fe centers in JDAC serve as effective hole and electron enrichment sites, effectively suppressing photoelectron recombination and enhancing photocurrent generation. Moreover, the enrichment of holes on Ni sites and electrons on Fe sites in JDAC facilitates the oxidation of Ni species to higher oxidation states under light illumination while promoting electron accumulation at Fe sites. This phenomenon satisfies the requirements for OER and ORR catalytic sites in electrocatalysis, thereby further facilitating the electrocatalytic reaction.

### Theoretical Analysis of JDAC

**Fig. 5:**
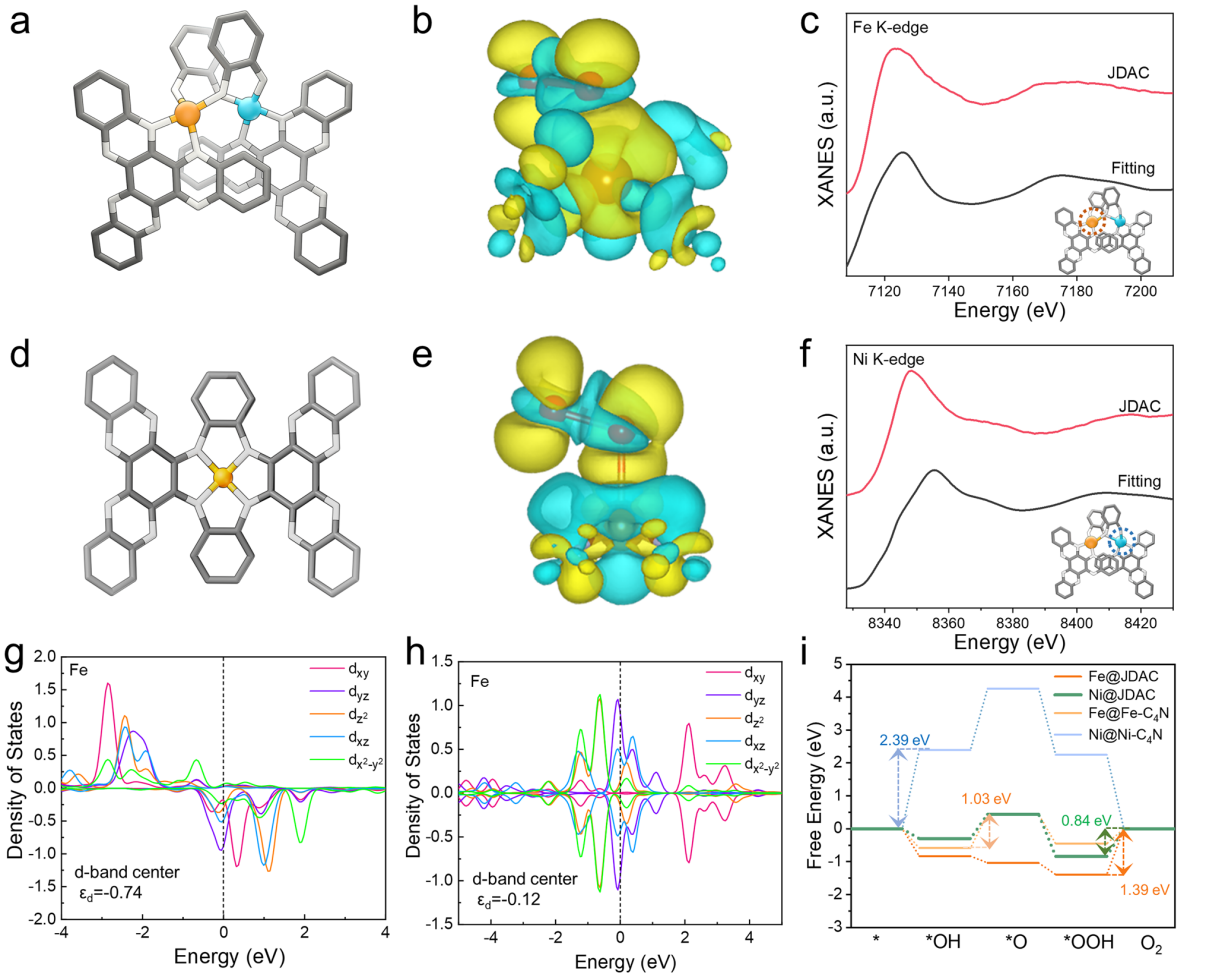
Simulated structure of **a** JDAC and **d** Fe-C_4_N. Electron distribution diagram of the active center with absorbed *OOH in **b** JDAC and **e** Fe-C_4_N. XANES fitting curves of the **c** Fe K-edge and **f** Ni K-edge of JDAC (fitting parameters are listed in Table S4). Density of states of Fe orbital in **g** JDAC and **h** Fe-C_4_N. **i** Calculated Gibbs free energy diagram of OER

First-principles calculations were carried out to gain deep insights into the role of JDAC in enhancing the oxygen electrocatalysis activities. The JDAC and Fe-C_4_N models containing M-N_4_ (M = Fe or Ni) sites anchored on C_4_N framework were constructed based on XAFS and NMR analysis results (Fig. 5a, d). The electron transfer process on the active site is distinctly depicted in the charge density distribution. The charge density difference plots reveal that the incorporation of Ni leads to an obvious increase in electron density around the Fe center (Fig. 5b, e). This reflects the neighboring Ni sites influence the adsorption behavior of *OOH on the Fe sites, thereby enhancing the oxygen redox kinetic process. XANES fitting results based on this model show alignment with experimental results, confirming the molecular structure of the JDAC catalyst (Fig. 5c, f). The density of states (DOS) of Fe 3*d* orbitals in JDAC and Fe-C_4_N was determined by density functional theory (DFT) calculations (Fig. 5g, h). As compared to Fe-C_4_N, 3*d* states of Fe in JDAC exhibit a diminished degree of localization, particularly for the *d*_xz_ and *d*_yz_ orbitals. Additionally, the *d*_z_^2^ orbital state of Fe crosses the Fermi level upon the incorporation of Ni atoms. This reconfiguration of Fe 3*d* states for JDAC can be attributed to the orbital interactions of dual atoms [62]. The incorporation of Ni atoms notably lowered the *d*-band center for Fe *d* states in JDAC (− 0.74 eV) compared to that of Fe-C_4_N (− 0.12 eV). This significant energetic discrepancy leads to a weaker bonding of Fe atoms with oxygen intermediates. Based on the OER and ORR four-electron reaction pathways, we calculated the Gibbs free energy diagrams for JDAC, Fe-C_4_N, and Ni-C_4_N (Figs. 5i and S35-S39). It is noted that both OER and ORR pathways on JDAC show more thermodynamically favorable as compared to Fe-C_4_N and Ni-C_4_N. In particular, the potential-determining steps (PDSs) for OER and ORR of JDAC are identified as the conversion of *OOH to O_2_ at the Ni site and the desorption of *OH at the Fe site, with overpotential of 0.84 and 0.83 eV, respectively. However, the PDS for OER at the Fe site of JDAC shows a notably high overpotential of 1.39 eV, while the PDS for ORR at the Ni site (1.28 eV) of JDAC is large. Thus, Ni and Fe sites are the dual-active centers for OER and ORR, respectively. The calculated results are in agreement with in situ XANES analysis and in situ Raman spectroscopy results. The PDS values of JDAC are significantly lower than those of Fe-C_4_N (1.03 V for OER and 0.89 eV for ORR) and Ni-C_4_N (2.39 eV for OER and 2.25 eV for ORR), indicating improved catalysis kinetics for OER/ORR in JDAC. Consequently, the integration of dual atoms in C_4_N modifies the energy levels of d orbitals in Fe sites, resulting in a lower *d*-band center, reduced intermediates binding strengths, and decreased oxygen redox reaction energy barriers.

### Light-Assisted Rechargeable Zn-Air Battery

**Fig. 6:**
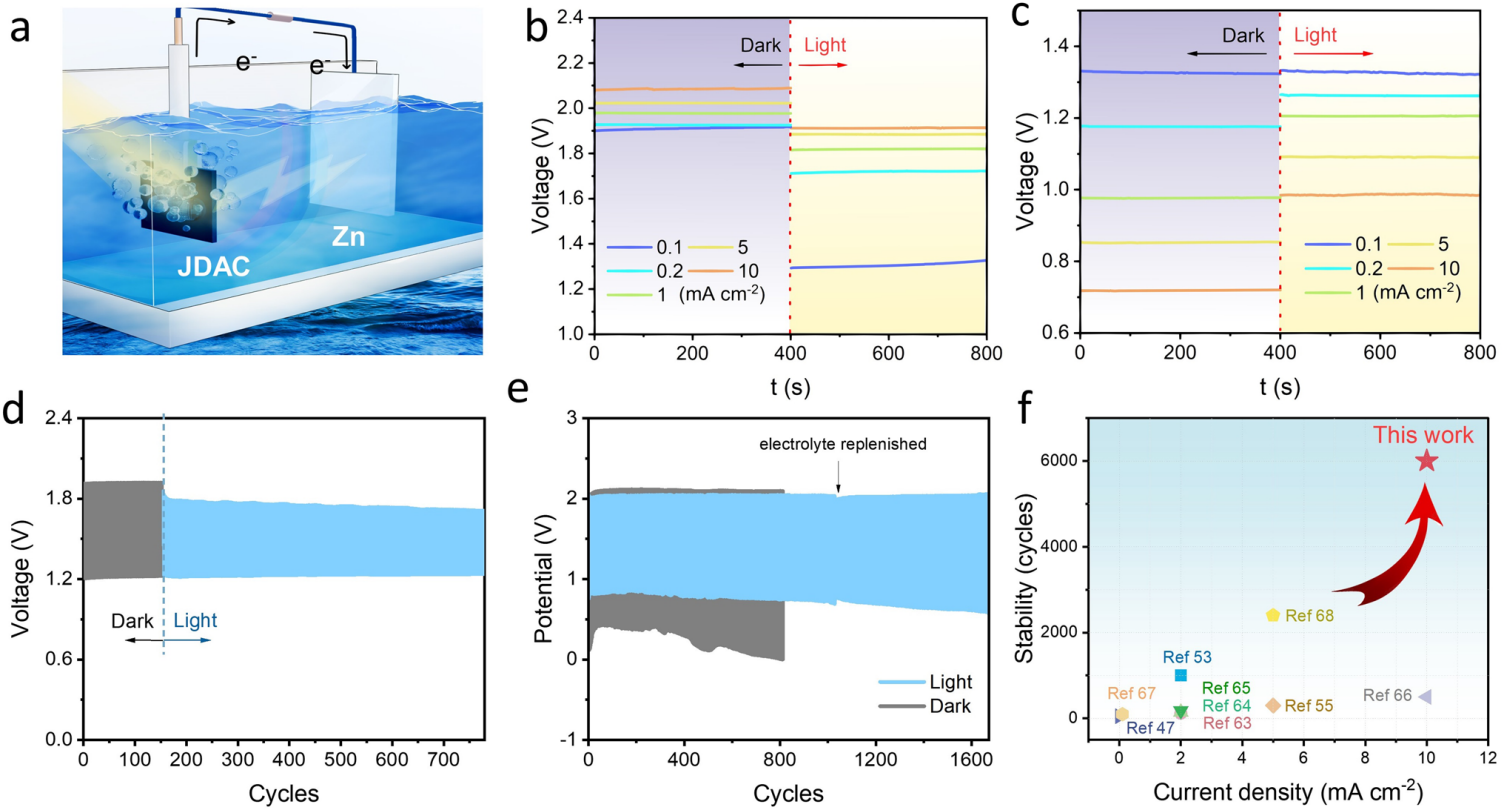
**a** Illustration of the light-assisted RZAB. **b** Charge and **c** discharge profile of RZAB under different currents with/without light illumination. **d, e** Charge/discharge cycles of light-assisted RZAB at **d** 1 mA cm^−2^ and **e** 20 mA cm^−2^. **f** Comparison of cycling stability of different samples [47, 53, 55, 63–68]

The JDAC catalysts was assembled into a RZAB with 6.0 M KOH and 0.2 M Zn(AC)_2_ and Zn foil as the electrolyte and anode, respectively, whereas an Xe lamp with an AM 1.5 G lens was employed as the simulated solar-light illumination (Fig. 6a). The charge and discharge potential of RZAB utilizing JDAC as the cathode was depicted as shown in Fig. 6b, c. At a current density of 0.1 mA cm^−2^, the charge potential can be significantly decreased by 620 mV upon illumination, corresponding to a 32% decrease. The discharge potential also shows an outstanding photo-induced increase of 267 mV at 10 mA cm^−2^, corresponding to an increase of 34%. This unique phenomenon is in accordance with LSV results in Fig. 4a, d. The JDAC-based RZAB can be operated at various current densities from 0.1 to 20 mA cm^−2^. The charge and discharge polarization curves exhibit that, under light illumination, the discharge voltages of the JDAC-based light-assisted RZAB notably increase to 1.23 V (1 mA cm^−2^) and 0.76 V (20 mA cm^−2^), while the charge voltage significantly decreases to 1.76 V (1 mA cm^−2^) and 2.03 V (20 mA cm^−2^) (Figs. S40 and S41). The narrower charge–discharge voltage gap (0.53 V at 1 mA cm^−2^ and 1.27 V at 20 mA cm^−2^) highlights the superior rechargeability and roundtrip efficiency. Under solar illumination, the charge voltage can be significantly reduced under 1 mA cm^−2^ (Fig. 6d). Notably, the light-assisted RZAB demonstrated remarkable stability at the current density of 10 and 20 mA cm^−2^, running for over 6000 and 1600 cycles, respectively (Figs. 6e and S42). This achievement surpasses other light-assisted ZABs that were previously reported (Fig. 6f and Table S5). Additionally, the JDAC-based light-assisted RZAB can operate over 300 cycles at a large current density of 50 mA cm^−2^ under illumination (Fig. S43). This demonstrates the potential of the JDAC-based light-assisted RZAB to handle demanding power requirements with high current densities.

## Conclusions

In summary, through the incorporation of Ni, Fe dual atoms in C_4_N framework, we successfully optimized the light-absorption capacity and carrier separation efficiency of photocatalysis, as well as enhanced the electrical conductivity and active site configuration of electrocatalysis catalysts. This integration resulted in the construction of catalytic electrodes with outstanding electrochemical and photoelectric conversion performance. The JDAC-based light-assisted RZABs can operate at high current density with extraordinary stability (300 cycles at 50 mA cm^−2^, 1600 cycles at 20 mA cm^−2^, and 6000 cycles at 10 mA cm^−2^ with negligible voltage decay under light illumination). This study will open an avenue toward the rational design of Janus dual-atom photoelectrocatalysts that efficiently convert solar energy into electric and chemical energy, such as light-assisted metal–sulfur batteries and metal–N_2_ batteries.

## Supplementary Information

Below is the link to the electronic supplementary material.

Supplementary file1 (DOCX 4118 KB)
