# Supplementary material for: Engineering Bipolar Doping in a Janus Dual-Atom Catalyst for Photo-Enhanced Rechargeable Zn-Air Battery
Source: Nanomicro Lett. 2025 Mar 28;17:203. doi: 10.1007/s40820-025-01707-2 (PMC11953513; doi:10.1007/s40820-025-01707-2)
Supplement: Supplementary file 1 — Supplementary file1 (DOCX 4118 KB) [file 40820_2025_1707_MOESM1_ESM.docx]

Supporting Information for

**Engineering Bipolar Doping in a Janus Dual-Atom Catalyst for Photo-Enhanced Rechargeable Zn-Air Battery**

Ning Liu^1^, Yinwu Li^1^, Wencai Liu^1^, Zhanhao Liang^1^, Bin Liao^1^, Fang Yang^2^, Ming Zhao^3^, Bo Yan^1^, Xuchun Gui^4^, Hong Bin Yang^3^, Dingshan Yu^2^*, Zhiping Zeng^1^*, Guowei Yang^1^

^1^State Key Laboratory of Optoelectronic Materials, School of Materials Science and Engineering, Sun Yat-sen University, Guangzhou 510275, P. R. China

^2^Key Laboratory for Polymeric Composite and Functional Materials of Ministry of Education, Key Laboratory of High-Performance Polymer-Based Composites of Guangdong Province, School of Chemistry, Sun Yat-Sen University, Guangzhou 510275, P. R. China

^3^School of Materials Science and Engineering, Suzhou University of Science and Technology, Suzhou 215009, P. R. China

^4^State Key Laboratory of Optoelectronic Materials and Technologies, School of Electronics and Information Technology, Sun Yat-sen University, Guangzhou 510275, P. R. China

*Corresponding authors. E-mail: [zzhip8@mail.sysu.edu.cn](mailto:zzhip8@mail.sysu.edu.cn) (Zhiping Zeng); [yudings@mail.sysu.edu.cn](mailto:yudings@mail.sysu.edu.cn) (Dingshan Yu)

**Supplementary Figures and Tables**


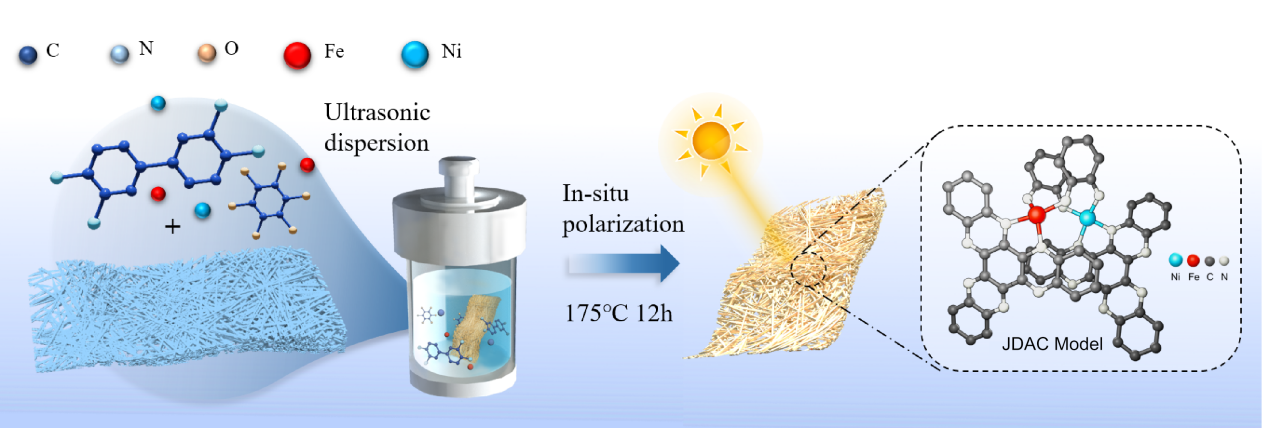


**Fig. S1** Schematic illustration of the preparation of JDAC


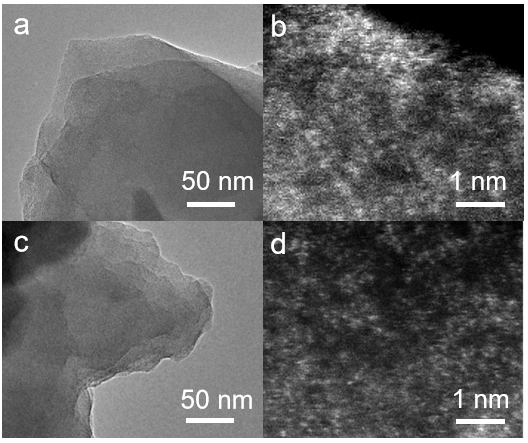


**Fig. S2** TEM images of (**a, b**) Fe-C_4_N, (**c, d**) Ni-C_4_N

~~
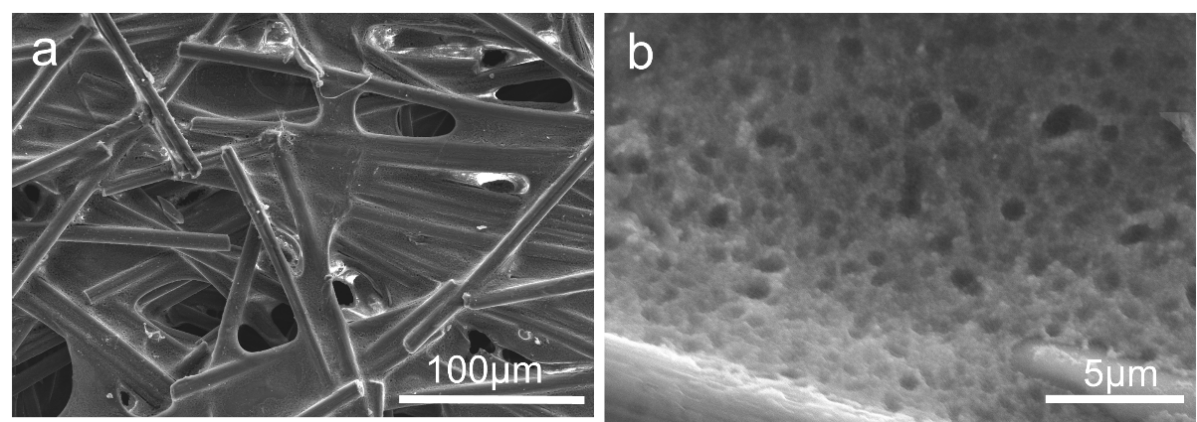
~~

**Fig. S3** SEM images of JDAC in-situ grown on carbon paper (**a**) Scale bar: 100 μm, (**b**) 5 μm


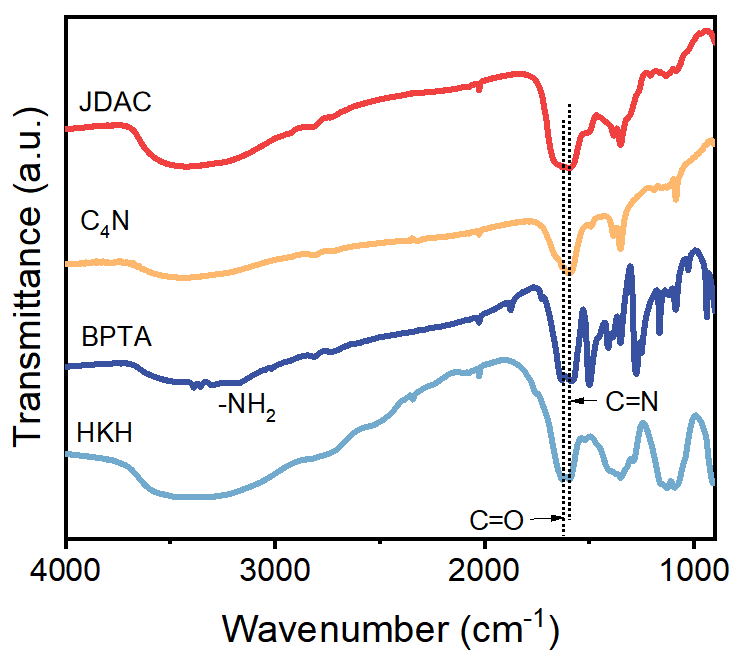


**Fig. S4** FT-IR spectra of precursors, C_4_N and JDAC


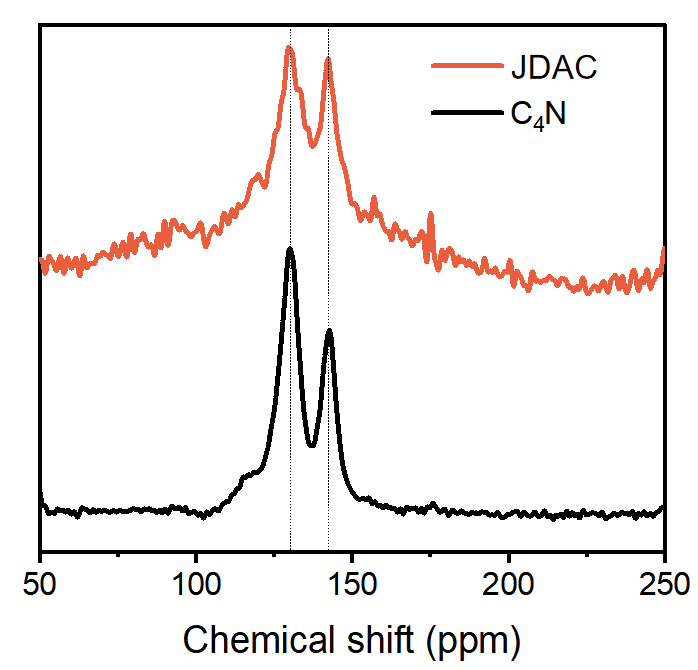


**Fig. S5** Solid-state ^13^C NMR spectrum of JDAC and C_4_N


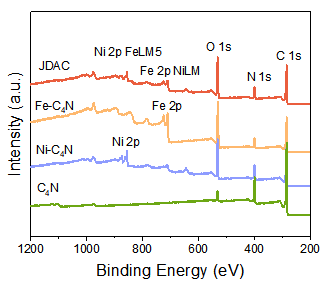


**Fig. S6** XPS spectra of JDAC, Fe-C_4_N, Ni-C_4_N, and C_4_N


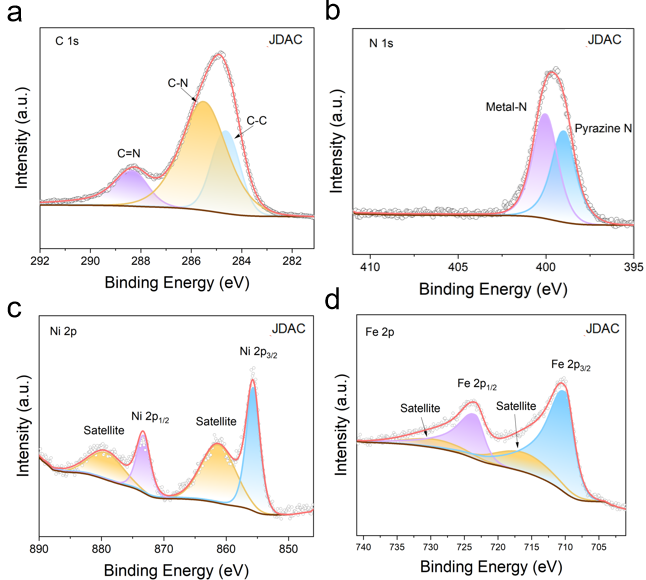


**Fig. S7** High-resolution XPS spectra of JDAC regarding (**a**) C *1s*, (**b**) N *1s*, (**c**) Ni *2p*, and (**d**) Fe *2p*

Note: The peaks of the configurations in JDAC are located at C=N (288.3 eV), C-N (285.5 eV), C-C (284.6 eV), metal-N (400.0 eV), pyrazine N (398.8 eV), Ni 2*p*_1/2_ (873.5 eV), Ni 2*p*_3/2_ (855.6 eV), Fe 2*p*_1/2_ (723.9 eV) and Fe 2*p*_3/2_ (710.2 eV), respectively.


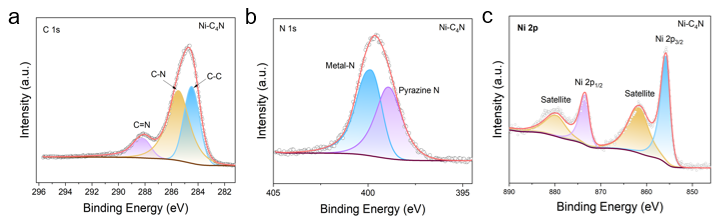


**Fig. S8** High-resolution XPS spectra of Ni-C_4_N regarding (**a**) C *1s*, (**b**) N *1s*, and (**c**) Ni *2p*

Note: The peaks of the configurations in Ni-C_4_N are located at C=N (288.2 eV), C-N (285.5 eV), C-C (284.6 eV), metal-N (400.0 eV), pyrazine N (398.8 eV), Ni 2*p*_1/2_ (873.5 eV), and Ni 2*p*_3/2_ (855.8 eV), respectively.


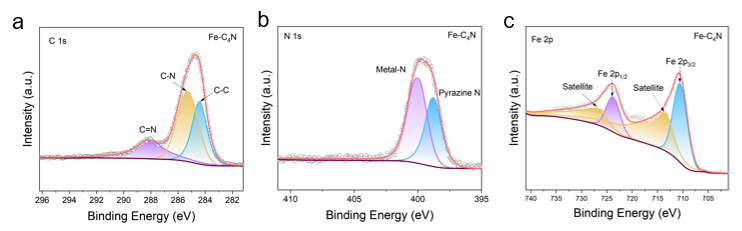


**Fig. S9** High-resolution XPS spectra of Fe-C_4_N regarding (**a**) C *1s*, (**b**) N *1s*, and (**c**) Fe *2p*

Note: The peaks of the configurations in Fe-C_4_N are located at C=N (288.3 eV), C-N (285.5 eV), C-C (284.6 eV), metal-N (400.0 eV), pyrazine N (398.8 eV), Fe 2*p*_1/2_ (723.9 eV) and Fe 2*p*_3/2_ (710.5 eV), respectively.


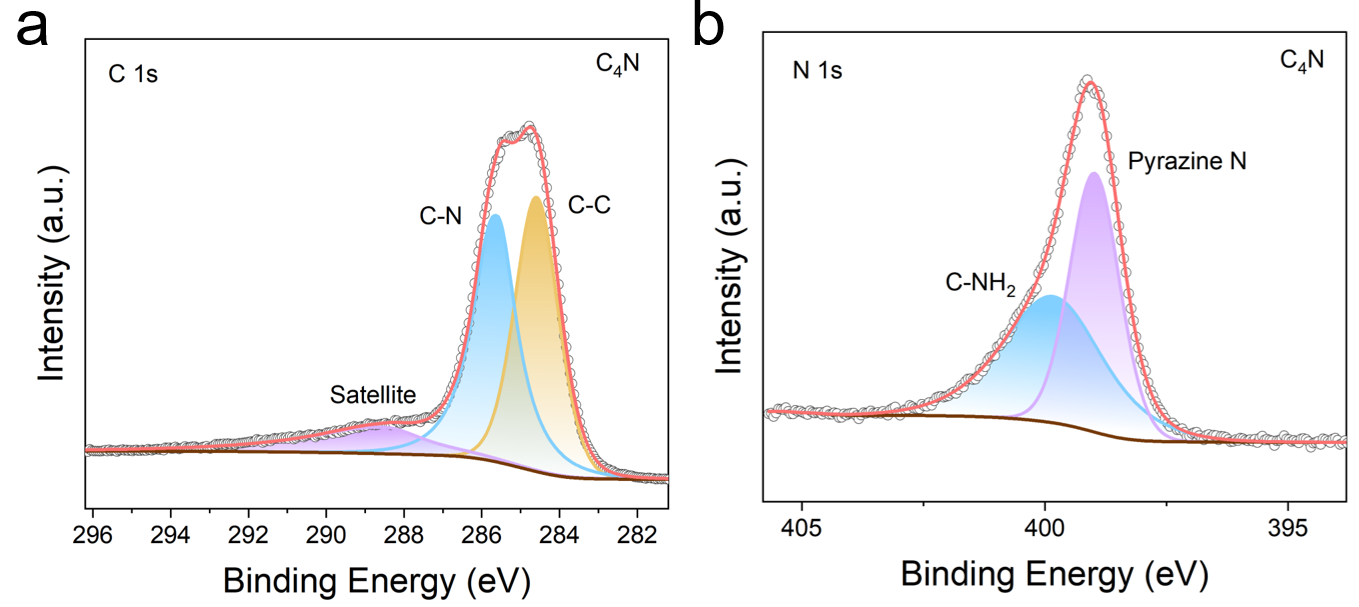


**Fig. S10** High-resolution XPS spectra of C_4_N regarding (**a**) C *1s* and (**b**) N *1s*

Note: The peaks of the configurations in C_4_N are located at C-N (285.5 eV), C-C (284.6 eV), residual -NH_2_ groups (399.9 eV), pyrazine N (398.8 eV), respectively.


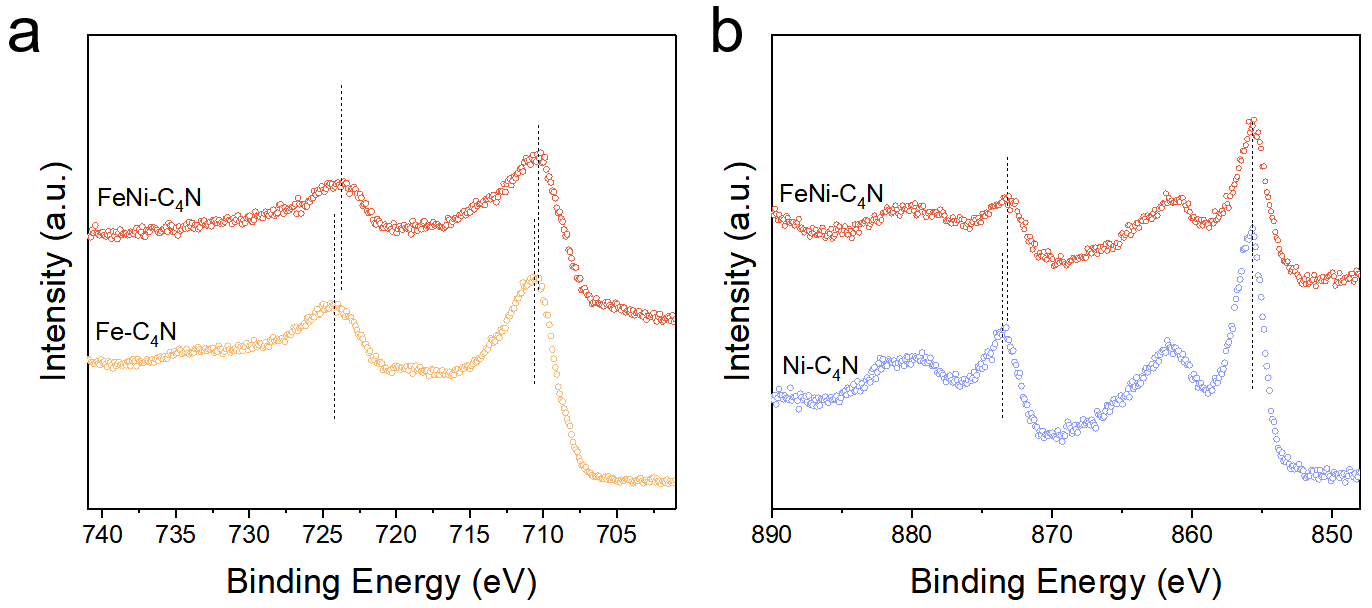


**Fig. S11** High resolution XPS survey of (**a**) Fe *2p* in JDAC and Fe-C_4_N, (**b**) Ni *2p* in JDAC and Ni-C_4_N

Note: The peaks positions of Fe *2p* in JDAC shows a negative shift from 724.1 eV to 723.4 eV, and from 710.8 eV to 710.7 eV compared to Fe-C_4_N. The peaks positions of Ni *2p* in JDAC also shows a negative shift from 873.5 eV to 873.4 eV, and 855.9 eV to 855.8 eV compared to Ni-C_4_N.


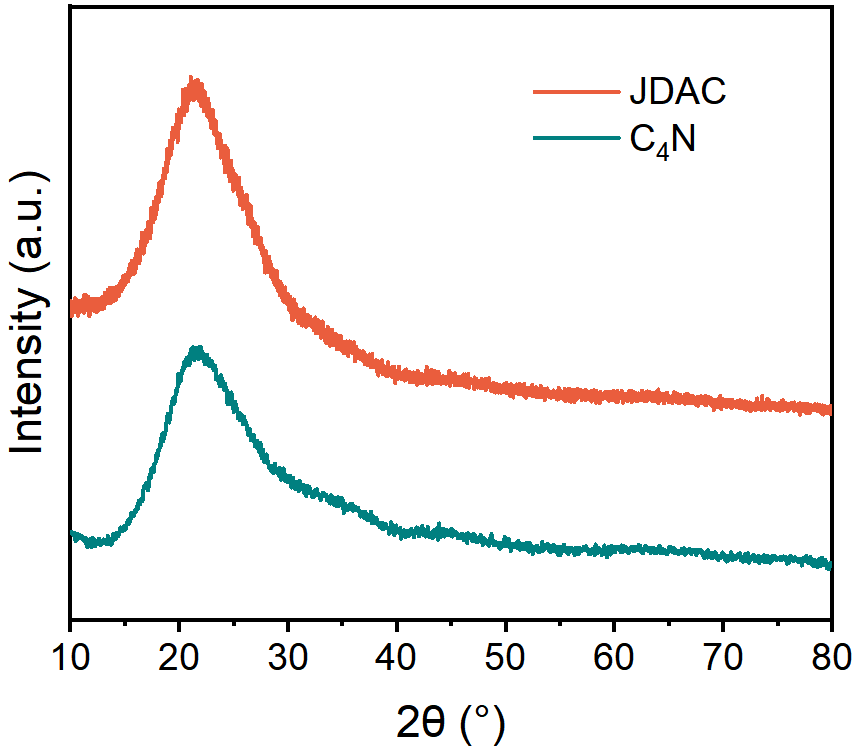


**Fig. S12** XRD patterns of JDAC and C_4_N


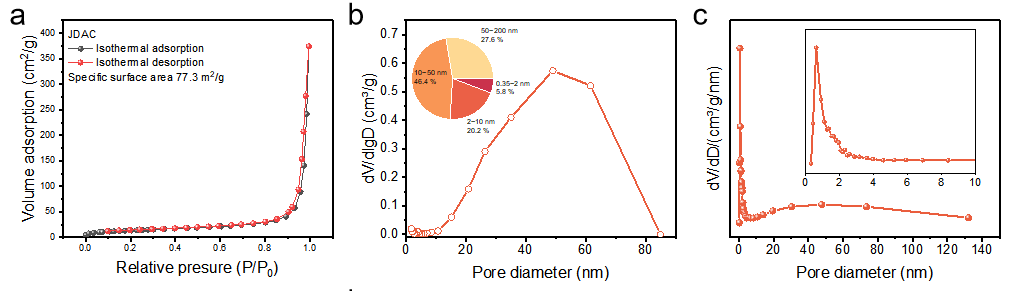


**Fig. S13** (**a**) Nitrogen adsorption isotherms measured at 77 K of JDAC. (**b**) BJH (desorption) pore volume and pore size logarithm curve (inset: pore size distribution) and (**c**) differential integral pore volume pore size distribution by NLDFT of JDAC


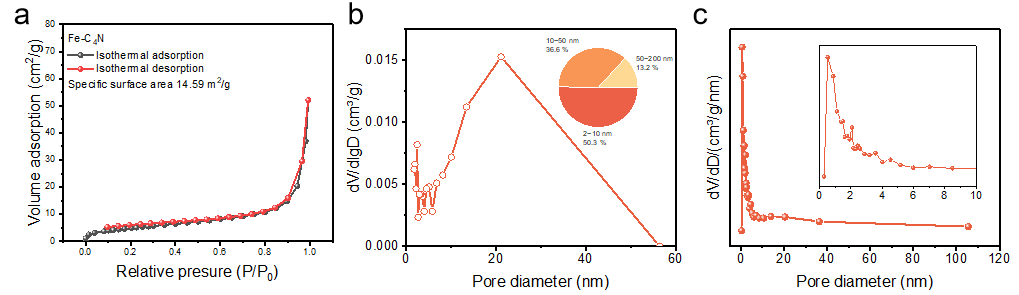


**Fig. S14** (**a**) Nitrogen adsorption isotherms measured at 77 K of Fe-C_4_N. (**b**) BJH (desorption) pore volume and pore size logarithm curve (inset: pore size distribution), and (**c**) differential integral pore volume pore size distribution by NLDFT of Fe-C_4_N


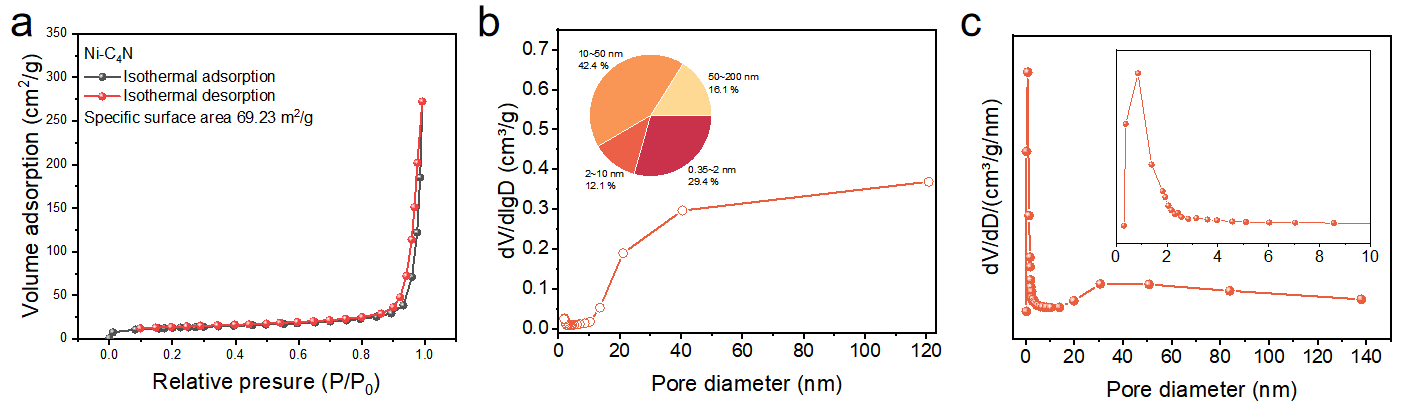


**Fig. S15** (**a**) Nitrogen adsorption isotherms measured at 77 K of Ni-C_4_N. (**b**) BJH (desorption) pore volume and pore size logarithm curve (inset: pore size distribution), and (**c**) differential integral pore volume pore size distribution by NLDFT of Ni-C_4_N


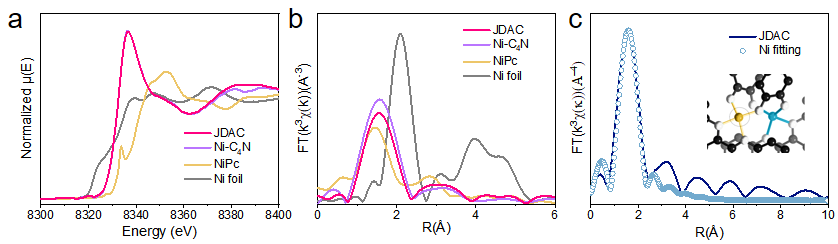


**Fig. S16** (**a**) XANES spectra of Ni k-edge. (**b**) Fourier transformation-EXAFS of Ni. (**c**) EXAFS fitting curves of Ni


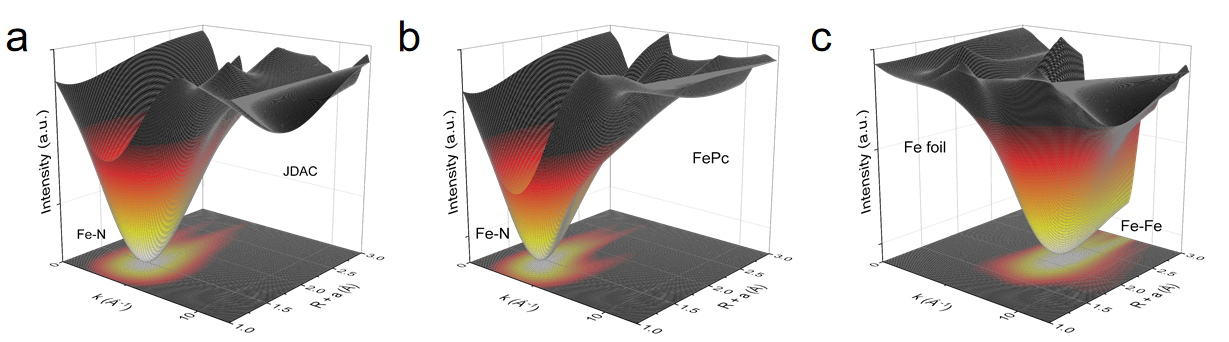


**Fig. S17** Wavelet transform -EXAFS of Fe k-edge in (**a**) JDAC, (**b**) FePc,and (**c**) Fe foil


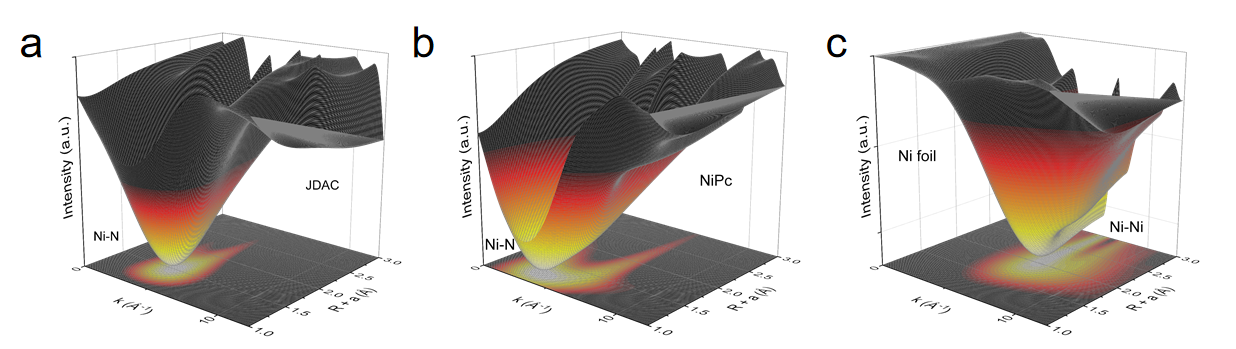


**Fig. S18** Wavelet transform -EXAFS of Ni k-edge in (**a**) JDAC, (**b**) NiPc, and (**c**) Ni foil


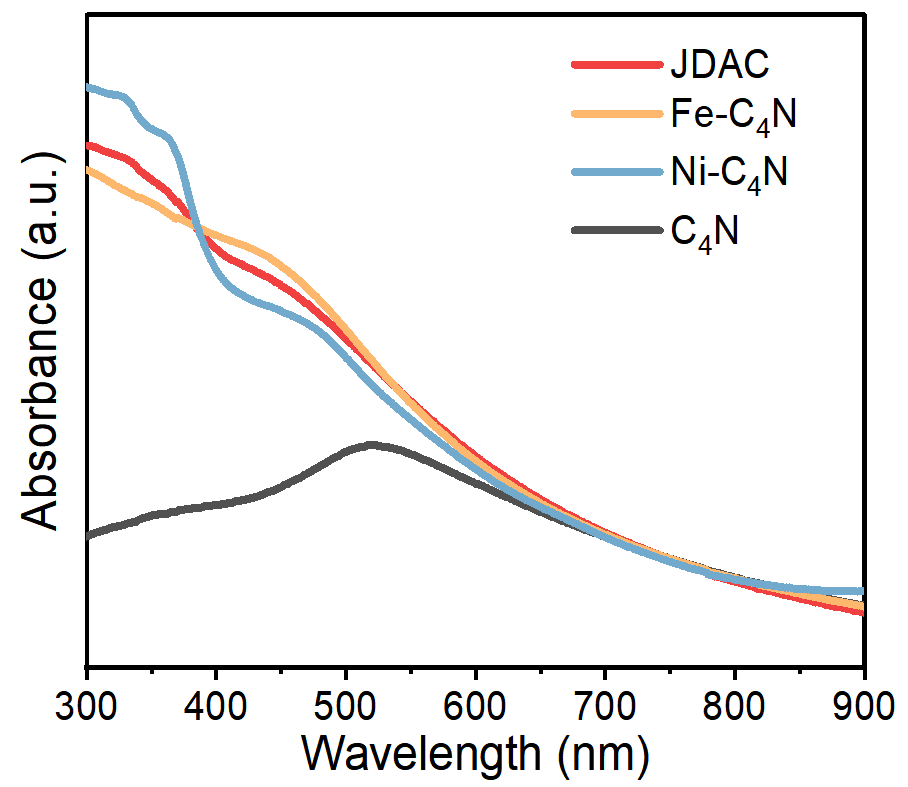


**Fig. S19** UV-Vis absorption spectrum of JDAC, Fe-C_4_N, Ni-C_4_N and C_4_N


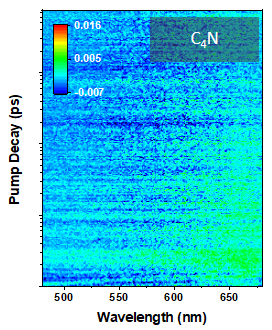


**Fig.** **S20** Femtosecond transient absorption spectroscopy (fs-TAS) of C_4_N


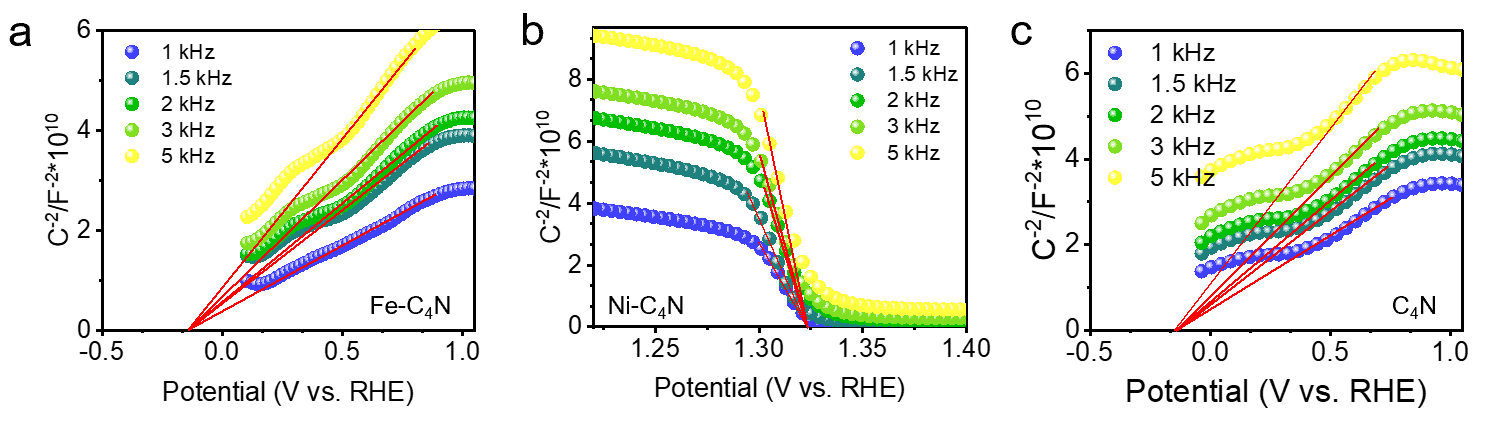


**Fig. S21** Mott-Schottky plots of C_scL_^-2^ *vs.* E of (**a**) Fe-C_4_N, (**b**) Ni-C_4_N, and (**c**) C_4_N


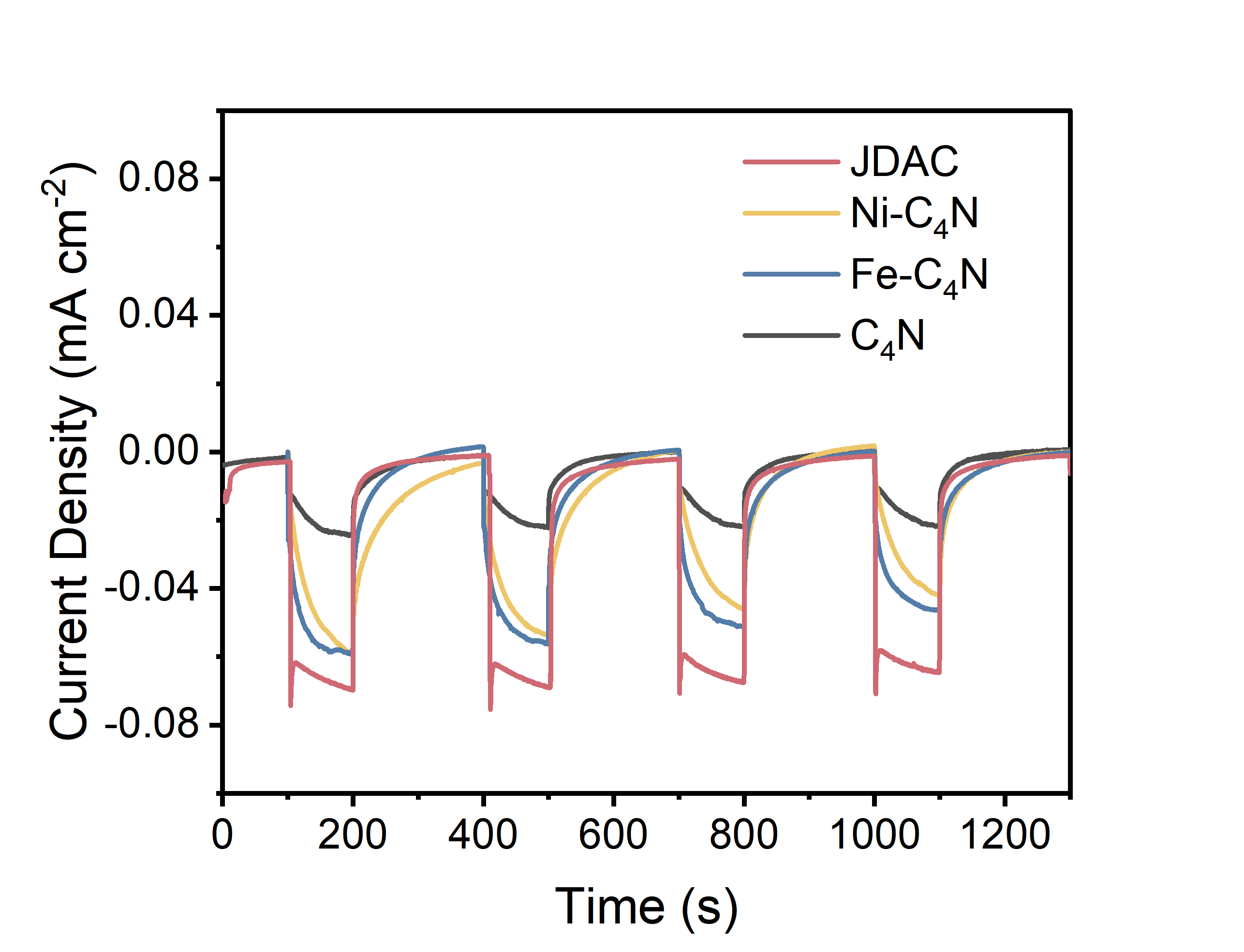


**Fig. 22** Comparison of the photocurrent of JDAC, Fe-C_4_N, Ni-C_4_N, and C_4_N


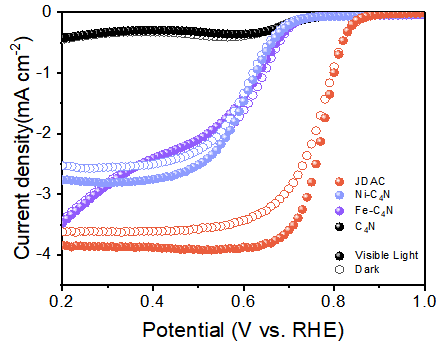


**Fig. S23** Linear sweep voltammograms of JDAC, Ni-C_4_N, Fe-C_4_N and C_4_N with and without visible light


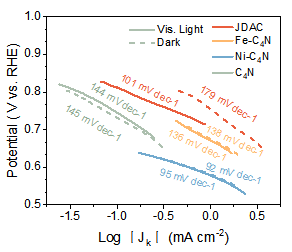


**Fig. S24** Tafel slopes for oxygen reduction reaction (ORR)


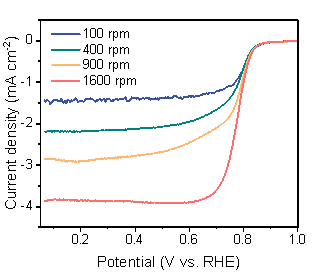


**Fig. S25** Linear sweep voltammograms curves of JDAC at 100 rpm, 400 rpm, 900 rpm and 1600 rpm under 420 cutoff visible light illumination


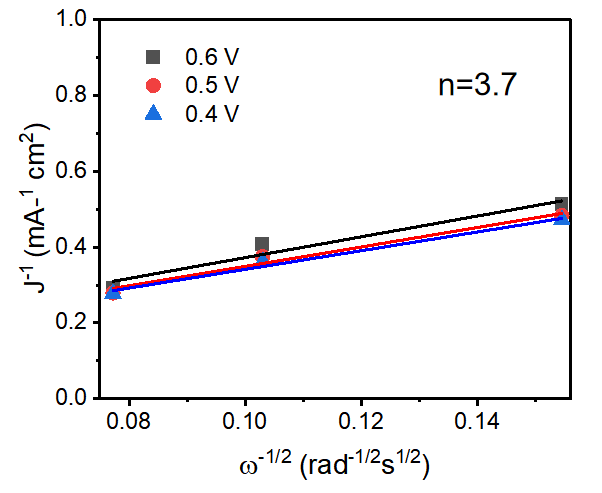


**Fig. S26** Koutecký-Levich plots of JDAC


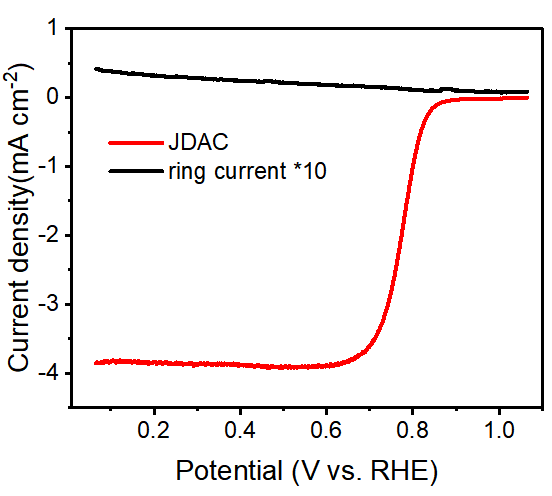


**Fig. S27** Rotating ring disk electrode measurements of JDAC under visible light illumination


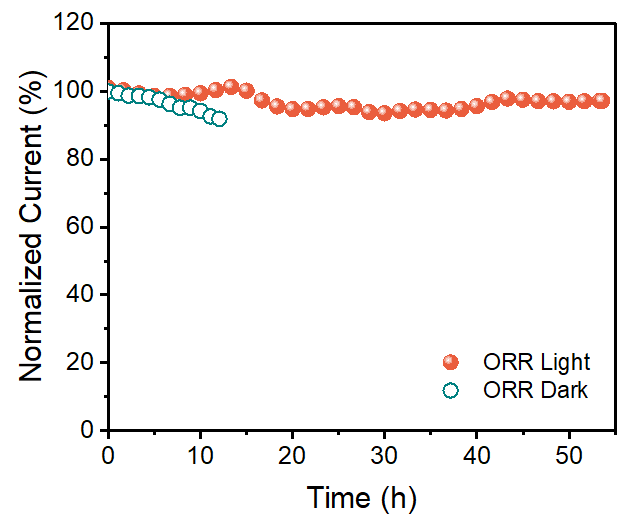


**Fig. S28** Stability measurement of JDAC in 0.1 M KOH solution with saturated O_2_ and under a bias voltage of 0.5 V (vs. RHE)


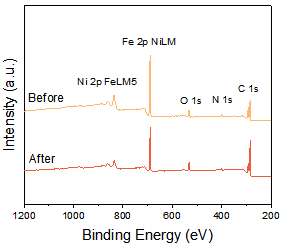


**Fig. S29** XPS spectra of JDAC before and after cycling tests


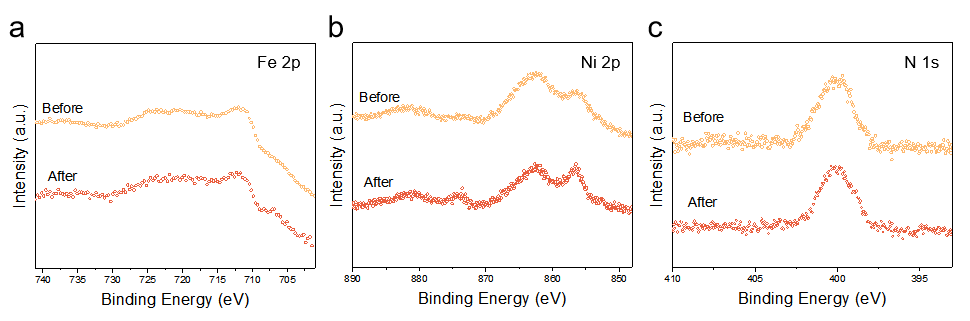


**Fig. S30** High-resolution XPS spectra of (**a**) Fe *2p*, (**b**) Ni *2p*, and (**c**) N *1s* of JDAC before and after cycling tests


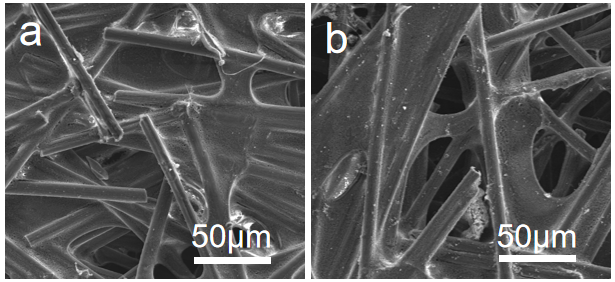


**Fig. S31** Comparison of the FESEM images of JDAC on carbon paper **a**) before and **b**) after stability measurements at 10 mA cm^-2^ for 1000 cycles


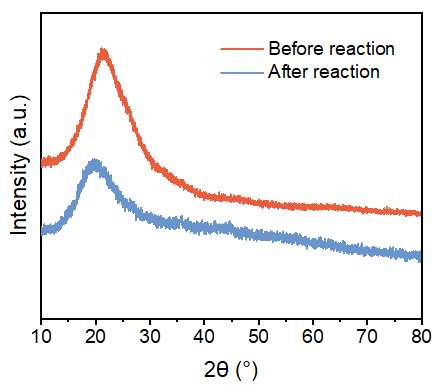


**Fig. S32** Comparison of the XRD spectra of JDAC before and after stability measurements at 10 mA cm^-2^ for 1000 cycles


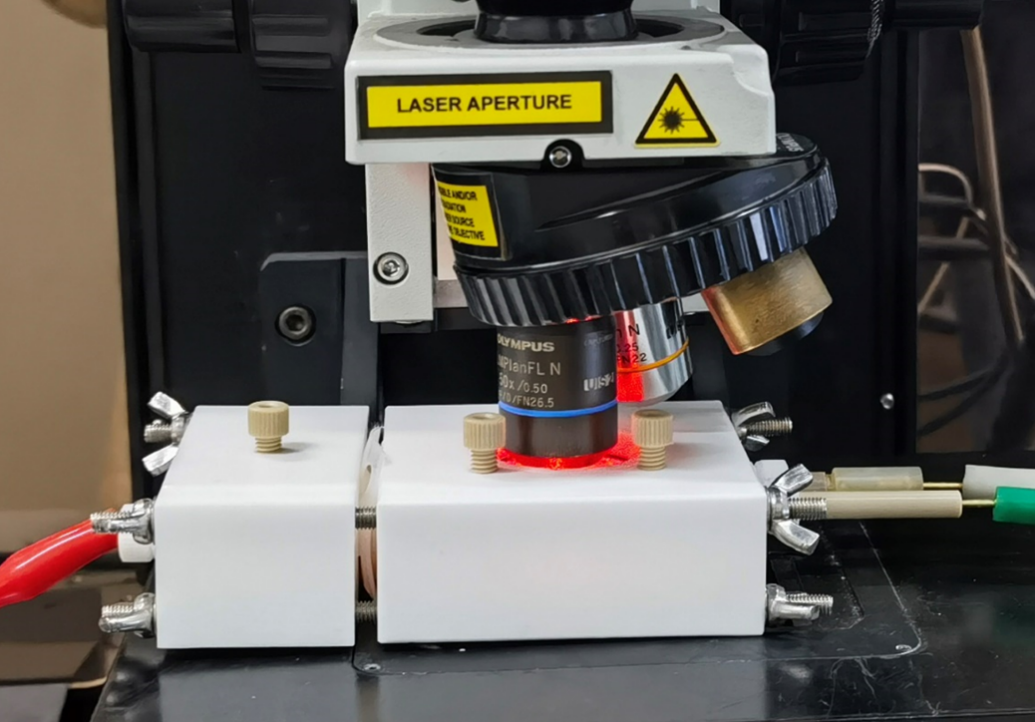


**Fig. S33** The digital photograph of the setup for in-situ Raman spectroscopy measurement


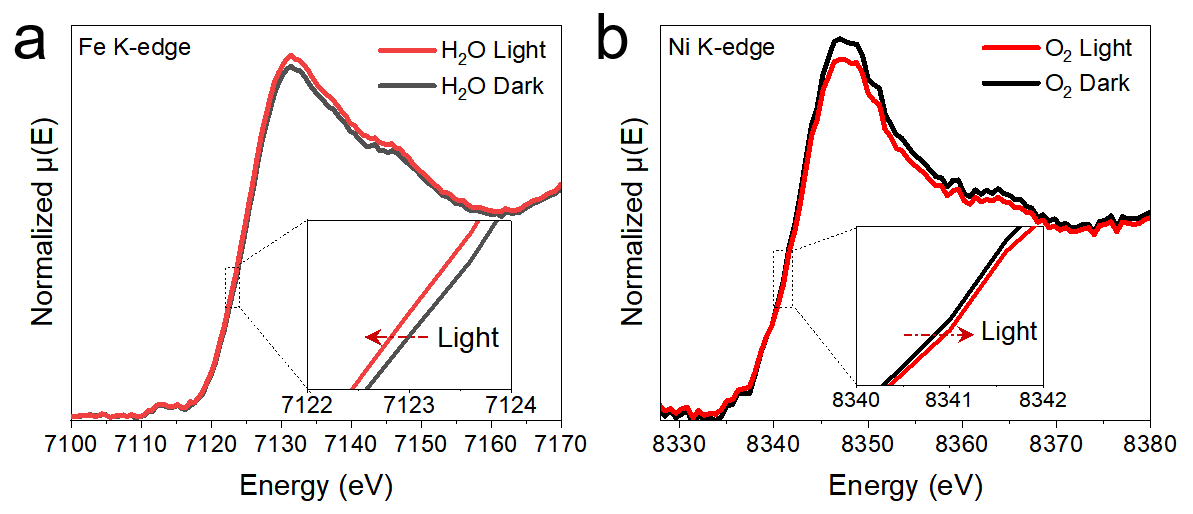


**Fig. S34** In-situ XANES with or without visible-light illumination of (a) Fe k-edge in stimulated OER environment with H_2_O and (b) Ni k-edge in stimulated ORR environment with O_2_


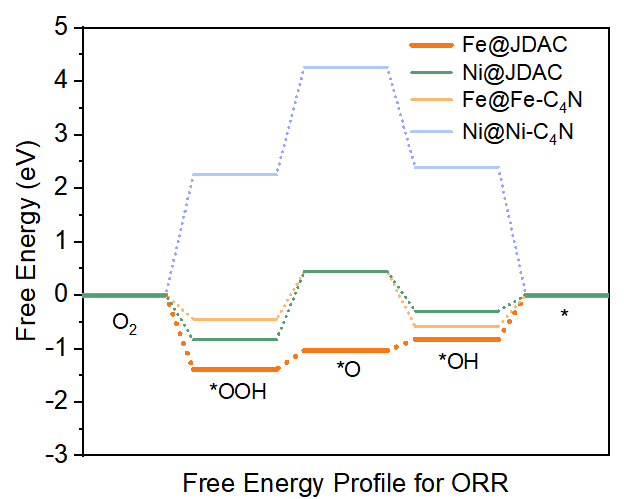


**Fig. S35** Calculated Gibbs free energy diagram of ORR

**Fig. S36** Free energy diagrams for ORR of JDAC without bias voltage


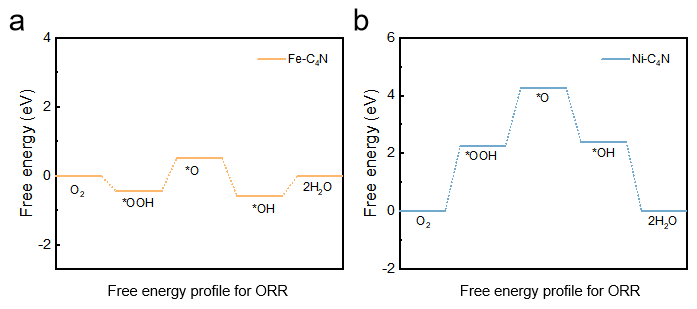


**Fig. S37** Free energy diagrams for ORR of (**a**) Fe-C_4_N, and (**b**) Ni-C_4_N


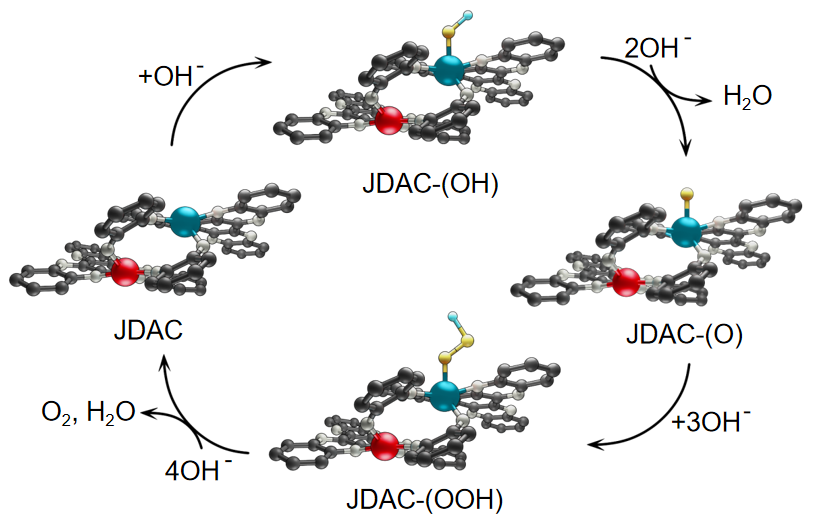


**Fig. S38** The reaction pathways of JDAC for OER. Blue, red, white, and grey balls are Ni, Fe, N, and C atoms, respectively


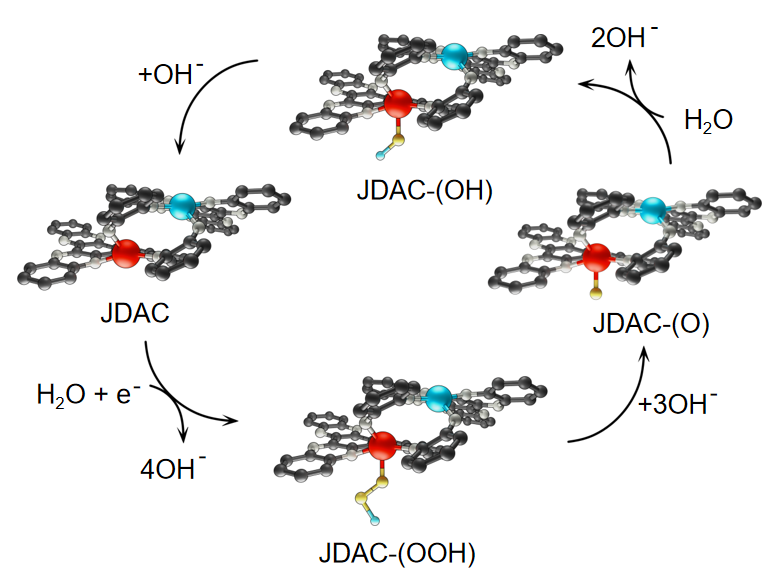


**Fig. S39** The reaction pathways of JDAC for ORR. Blue, red, white, and grey balls are Ni, Fe, N, and C atoms, respectively


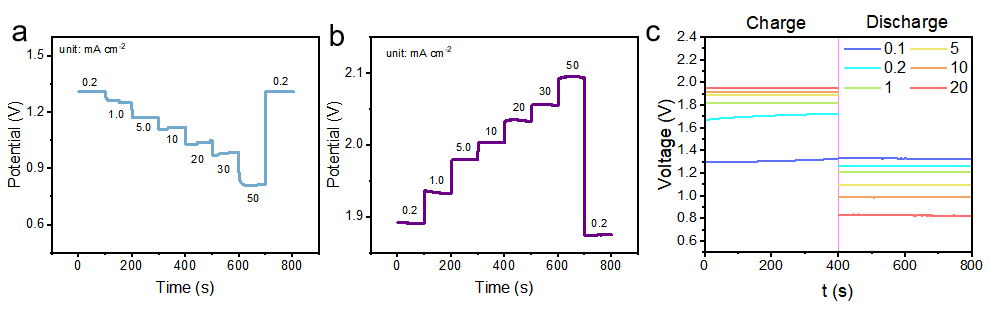


**Fig. S40** (**a**) Galvanostatic discharge and (**b**) galvanostatic charge curves of RZAB at different current densities under AM 1.5 G illumination


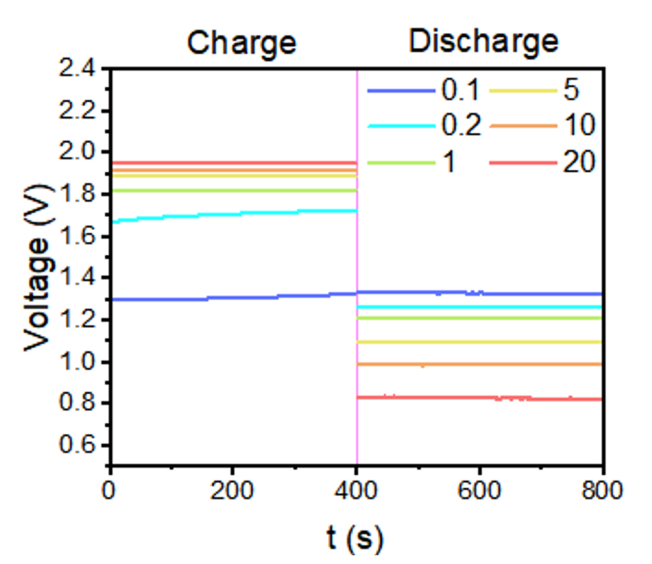


**Fig. S41** Comparison of galvanostatic charge/discharge curves of RZAB under AM 1.5 G illumination


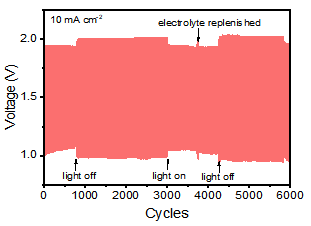


**Fig. S42** Galvanostatic discharge-charge cycling curves with/without AM 1.5 G illumination 10 mA cm^-2^


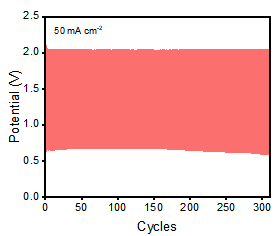


**Fig. S43** Galvanostatic discharge-charge cycling curves under AM 1.5 G at 50 mA cm^-2^

**Table S1** Elemental percentage for the catalysts based on the ICP-AES test

| Sample | Fe (wt%) | Ni (wt%) |
| --- | --- | --- |
| JDAC | 7.96 | 6.52 |

**Table S2** Parameters of EXAFS fittings for the catalysts


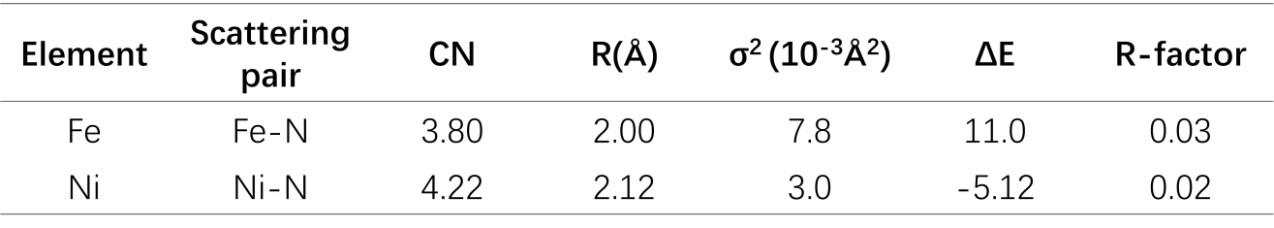


**Table S3** Electrocatalysis performances of photo-assisted bifunctional oxygen catalysis

| **Bifunctional air cathode** | **Light source** | **Overpotential** | **Onset potential** | **ΔE** | **Ref** |
| --- | --- | --- | --- | --- | --- |
| **JDAC** | **visible light** | **170** | **1.031** | **0.62** | **This work** |
| CZ |  | 410 | 0.8 | 0.84 | [S1] |
| CaFeRuO_6_ (CFR) | visible light | 400 | 0.9 | 0.85 | [S2] |
| CoFe_2_O_4_-N,S-C | 300W Xe lamp | 410 | 0.92 | 0.8 | [S3] |
| ZnO/Cu_2_O (ZnO/CuO) | visible light | - | 0.9 | - | [S4] |
| TiO_2_@In_2_Se_3_@Ag_3_PO_4_ | 365 nm | -510 | 1.32 | -0.6 | [S5] |
| polytrithiophene (pTTh) | 365 nm | N.A. | 1.4 | - | [S6] |
| MnCo_2_O_4_ | visible light | ~310 | 0.87 | - | [S7] |
| NG/CCN | visible light | 300 | 0.77 | 0.76 | [S8] |
| pTTh | visible light | 760 | 0.91 | 1.16 | [S9] |
| Ni_12_P_5_@NCNT | AM 1.5G | 360 | 0.9 | - | [S10] |
| C_4_N@TiO_2_NR | 395 nm | ~140 | - | - | [S11] |
| C_4_N |  | N.A. | 0.8 | - | [S12] |
| FeNi-S,N-HCS |  | 380 | - | 0.79 | [S13] |

**Tabel S4** Fitting parameters of the near-edge simulated spectra of Fe and Ni

| Element | Radius | Ecent | Elarg | Gamma_max | Gamma_hole | E_Cut |
| --- | --- | --- | --- | --- | --- | --- |
| Fe | 4 | 15 | 20 | 4 | 5 | 1.5 |
| Ni | 6.5 | 10 | 3.18 | 6.95 | 3 | 1.5 |

**Table S5** Performance comparison of photo-assisted liquid RZABs

| Bifunctional air cathode | Current (mA cm^-2^ ) | Charge Voltage (V) | Discharge Voltage (V) | Voltage Gap (V) | Stability | Refs |
| --- | --- | --- | --- | --- | --- | --- |
| **JDAC** | **1** | **1.81** | **1.21** | **0.60** |  | **This work** |
|  | **5** | **1.88** | **1.09** | **0.79** |  |  |
|  | **10** | **1.91** | **0.99** | **0.92** | **6000 cycles** |  |
|  | **20** | **1.95** | **0.83** | **1.12** | **1688 cycles** |  |
|  | **50** | **2.03** | **0.67** | **1.36** | **338 cycles** |  |
| CZ | 2 | 1.99 | 1.18 | 0.81 | 1000 cycles@2mA cm^-2^ | [S1] |
| CZ | 5 | 2.01 | 1.11 | 0.9 | 334 hrs. @ 5 mA cm^-2^ | [S1] |
| 1@ZIF-67 | 2 | 2 | 1.15 | ~0.85 | ~50%@110 hrs. | [S14] |
| Co@NPCFs | 5 | 2.2 | 1.15 | ~1.05 | 80 hrs. | [S15] |
| FeCo–C/N | 2 | 2.05 | 1.15 | ~0.9 | 180 cycles 60 hrs. | [S16] |
| ZIF-67@Pt/CB | 5 | 2.08 | 1.17 | 0.91 | 50 hrs. | [S17] |
| Spinel Co_3_O_4_ | 2 | 2.01 | 1.19 | 0.82 | 70 hrs. | [S18] |
| Co-N-CNTs | 2 | ~2.30 | ~1 | ~1.30 | 145 cycles 16 hrs. | [S19] |
| CoNiFe-S MNs | 2 | 1.88 | 1.12 | 0.76 | 120 cycles 40 hrs. | [S20] |
| NiFe_2_O_4_/FeNi_2_S_4_ HNSs | 2 | ~2.10 | ~0.98 | 1.2 | 125 hrs. | [S21] |
| CNF@Zn/CoNC | 2 | 2.1 | 1.19 | 0.91 | 150 hrs. | [S22] |
| BNPC-1100 | 2 | 2.2 | 1.14 | 1.06 | 100 hrs. | [S23] |
| CoFe_20_@CC | 5 | 1.10 – 2.10 |  | ~1.00 | 130 hrs. | [S24] |
| Fe/Co-N/P-9 | 5 | 0.9 | 2.05 | ~1.05 | 130 cycles | [S25] |
| CoFe_2_O_4_-N,S-C | 5 | ~2.04 | 1.19 | ~0.85 | 300 cycles @ 5mA cm^-2^ | [S3] |
| ZnO/Cu2O (ZnO/CuO) | 0.1 | ~1.5 | 1.28 | ~0.22 | 22 hours @ 0.1 mA cm^-2^ | [S4] |
| polytrithiophene (pTTh) | 0.1 | ~1.98 | ~1.78 | 0.2 | 64 hours @ 0.1 mA cm^-2^ | [S6] |
| pTTh | 0.1 | 1.81 | 1.22 | 0.6 | 380 hours @ 0.1 mA cm^-2^ | [S9] |
| Ni_12_P_5_@NCNT | 1 | 1.94 | 1.19 | 0.75 | 500 cycles @ 10 mA cm^-2^ | [S10] |
| αFe_2_O_3_ | 0.5 | ~1.64 | ~1.15 | 0.49 | 50 hours @ 0.5 mA cm^-2^ | [S14] |
| C4N | 0.01 | 1.35 | 1.32 | 0.03 | 50 cycles @ 0.01 mA cm^-2^ | [S12] |
| FeNi-S,N-HCS | 5 | ~2.0 | ~1.1 | ~0.9 | 120 hours @ 5 mA cm^-2^ | [S13] |

**Supplementary References**

1. R. Ren, G. Liu, J.Y. Kim, R.E.A. Ardhi, M.X. Tran et al., Photoactive g-C_3_N_4_/CuZIF-67 bifunctional electrocatalyst with staggered p-n heterojunction for rechargeable Zn-air batteries. Appl. Catal. B Environ. **306**, 121096 (2022). <https://doi.org/10.1016/j.apcatb.2022.121096>
2. N. Kumar, K. Naveen, M. Kumar, T. C. Nagaiah, R. Sakla et al., Multifunctionality exploration of Ca_2_FeRuO_6_: an efficient trifunctional electrocatalyst toward OER/ORR/HER and photocatalyst for water splitting. ACS Appl. Energy Mater. **4**, 1323–1334 (2021). <https://doi.org/10.1021/acsaem.0c02579>
3. Z. Xiao, X. Lv, S. Liu, Q. Liu, F. Wang et al., Electronic band structure engineering of transition metal oxide-N, S-doped carbon catalysts for photoassisted oxygen reduction and oxygen evolution catalysis. Adv. Mater. Interfaces **9**(1), 2101386 (2022). <https://doi.org/10.1002/admi.202101386>
4. D. Bu, M. Batmunkh, Y. Zhang, Y. Li, B. Qian et al., Rechargeable sunlight-promoted Zn-air battery constructed by bifunctional oxygen photoelectrodes: Energy-band switching between ZnO/Cu_2_O and ZnO/CuO in charge-discharge cycles. Chem. Eng. J. **433**, 133559 (2022). <https://doi.org/10.1016/j.cej.2021.133559>
5. H. Feng, C. Zhang, Z. Liu, J. Sang, S. Xue et al., A light-activated TiO_2_@In_2_Se_3_@Ag_3_PO_4_ cathode for high-performance Zn-Air batteries. Chem. Eng. J. **434**, 134650 (2022). <https://doi.org/10.1016/j.cej.2022.134650>
6. H. Feng, C. Zhang, Z. Liu, J. Sang, S. Xue et al., A light-activated TiO_2_@In_2_Se_3_@Ag_3_PO_4_ cathode for high-performance Zn-Air batteries. Chem. Eng. J. **434**, 134650 (2022). <https://doi.org/10.1016/j.cej.2022.134650>
7. C. Tomon, S. Sarawutanukul, S. Duangdangchote, A. Krittayavathananon, M. Sawangphruk, MnCo_2_O_4_ nanofibers as efficient photo-electrocatalyst for oxygen evolution reaction and oxygen reduction reaction. ECS Trans. **97**(7), 71–86 (2020). <https://doi.org/10.1149/09707.0071ecst>
8. Y. Yang, B. Hu, W. Zhao, Q. Yang, F. Yang et al., Bridging N-doped graphene and carbon rich C_3_N_4_ layers for photo-promoted multi-functional electrocatalysts. Electrochim. Acta **317**, 25–33 (2019). <https://doi.org/10.1016/j.electacta.2019.05.140>
9. Y. Yang, B. Hu, W. Zhao, Q. Yang, F. Yang et al., Bridging N-doped graphene and carbon rich C_3_N_4_ layers for photo-promoted multi-functional electrocatalysts. Electrochim. Acta **317**, 25–33 (2019). <https://doi.org/10.1016/j.electacta.2019.05.140>
10. J. Lv, S.C. Abbas, Y. Huang, Q. Liu, M. Wu et al., A photo-responsive bifunctional electrocatalyst for oxygen reduction and evolution reactions. Nano Energy **43**, 130–137 (2018). <https://doi.org/10.1016/j.nanoen.2017.11.020>
11. J. Lv, S.C. Abbas, Y. Huang, Q. Liu, M. Wu et al., A photo-responsive bifunctional electrocatalyst for oxygen reduction and evolution reactions. Nano Energy **43**, 130–137 (2018). <https://doi.org/10.1016/j.nanoen.2017.11.020>
12. Z. Fang, Y. Li, J. Li, C. Shu, L. Zhong et al., Capturing visible light in low-band-gap C_4_N-derived responsive bifunctional air electrodes for solar energy conversion and storage. Angew. Chem. Int. Ed. **60**(32), 17615–17621 (2021). <https://doi.org/10.1002/anie.202104790>
13. S. Zheng, M. Chen, K. Chen, Y. Wu, J. Yu et al., Solar-light-responsive zinc–air battery with self-regulated charge–discharge performance based on photothermal effect. ACS Appl. Mater. Interfaces **15**(2), 2985–2995 (2023). <https://doi.org/10.1021/acsami.2c19663>
14. Z. Liang, H. Guo, G. Zhou, K. Guo, B. Wang, H. Lei, W. Zhang, H. Zheng, U. Apfel, R. Cao, Metal–organic-framework-supported molecular electrocatalysis for the oxygen reduction reaction. Angew. Chem. Int. Ed. **133**, 8553-8557 (2021). <https://doi.org/10.1002/ange.202016024>
15. Y. Chen, W. Zhang, Z. Zhu, L. Zhang, J. Yang et al., Co nanoparticles combined with nitrogen-doped graphitic carbon anchored on carbon fibers as a self-standing air electrode for flexible zinc–air batteries. J. Mater. Chem. A **8**, 7184-7191 (2020). <https://doi.org/10.1039/D0TA00793E>
16. C. Zhang, H. Yang, D. Zhong, Y. Xu, Y. Wang et al., A yolk–shell structured metal–organic framework with encapsulated iron-porphyrin and its derived bimetallic nitrogen-doped porous carbon for an efficient oxygen reduction reaction. J. Mater. Chem. A **8**, 9536-9544 (2020). <https://doi.org/10.1039/D0TA00962H>
17. J. Li, Z. Meng, D.J.L. Brett, P.R. Shearing, N.T. Skipper et al., High-performance zinc–air batteries with scalable metal–organic frameworks and platinum carbon black bifunctional catalysts. ACS Appl. Mater. Interfaces **12**(38), 42696–42703 (2020). <https://doi.org/10.1021/acsami.0c10151>
18. C. Tomon, S. Sarawutanukul, S. Duangdangchote, A. Krittayavathananon, M. Sawangphruk, Photoactive Zn–air batteries using spinel-type cobalt oxide as a bifunctional photocatalyst at the air cathode. Chem. Commun. **55**(42), 5855–5858 (2019). <https://doi.org/10.1039/c9cc01876j>
19. T. Wang, Z. Kou, S. Mu, J. Liu, D. He et al., 2D dual-metal zeolitic-imidazolate-framework-(ZIF)-derived bifunctional air electrodes with ultrahigh electrochemical properties for rechargeable zinc–air batteries. Adv. Funct. Mater. **28**, 1705048 (2018). <https://doi.org/10.1002/adfm.201705048>
20. H. Yang, B. Wang, H. Li, B. Ni, K. Wang et al., Trimetallic sulfide mesoporous nanospheres as superior electrocatalysts for rechargeable Zn–air batteries. Adv. Energy Mater. **8**(34), 1801839 (2018). <https://doi.org/10.1002/aenm.201801839>
21. L. An, Z. Zhang, J. Feng, F. Lv, Y. Li et al., Heterostructure-promoted oxygen electrocatalysis enables rechargeable zinc–air battery with neutral aqueous electrolyte. J. Am. Chem. Soc. **140**, 17624-17631 (2018). <https://doi.org/10.1021/jacs.8b09805>
22. Y. Zhao, Q. Lai, J. Zhu, J. Zhong, Z. Tang et al., Controllable construction of core–shell Polymer@Zeolitic imidazolate frameworks fiber derived heteroatom-doped carbon nanofiber network for efficient oxygen electrocatalysis. Small **14**(19), 1704207 (2018). <https://doi.org/10.1002/smll.201704207>
23. Y. Qian, Z. Hu, X. Ge, S. Yang, Y. Peng et al., A metal-free ORR/OER bifunctional electrocatalyst derived from metal-organic frameworks for rechargeable Zn-Air batteries. Carbon **111**, 641-650 (2017). <https://doi.org/10.1016/j.carbon.2016.10.046>
24. J. Di, J. Guo, N. Wang, G. Ma, Multicomponent doped sugar-coated haws stick-like nanofibers as efficient oxygen reduction reaction catalysts for the Zn–air battery. ACS Sustainable Chem. Eng. **7**(8), 7716–7727 (2019). <https://doi.org/10.1021/acssuschemeng.8b06447>
25. C. Hou, L. Zou, Q. Xu, Hierarchical superstructures: a hydrangea-like superstructure of open carbon cages with hierarchical porosity and highly active metal sites. Adv. Mater. **31**, 1904689 (2019). <https://doi.org/10.1002/adma.201970327>
